# Sphingolipid–renal interplay in coronary artery calcification: ceramide profiling and imaging validation from the CRUISE-MET study

**DOI:** 10.3389/fendo.2026.1974775

**Published:** 2026-10-14

**Authors:** Qiang Chen, Sijia Guo, Xuexi Li, Yike Li, Yingying Xie, Sunjing Fu, Ruoyun Ma, Xiaxia Peng, Sheng Xie, Yanxiang Gao, Jingang Zheng

**Affiliations:** 1 Department of Cardiology, China-Japan Friendship Hospital, Chinese Academy of Medical Sciences, Peking Union Medical College, Beijing, China; 2 Department of Cardiology, The Third People's Hospital of Chengdu, The Affiliated Hospital of Southwest Jiaotong University, Chengdu, China; 3 Department of Radiology, China-Japan Friendship Hospital, Beijing, China; 4 Peking University China-Japan Friendship School of Clinical Medicine, Beijing, China; 5 Department of Cardiology, China-Japan Friendship Hospital, Beijing, China

**Keywords:** calcification, ceramide, chronic kidney disease, coronary artery disease, renal impairment

## Abstract

**Background:**

Coronary artery calcification (CAC) represents a distinct imaging phenotype reflecting advanced vascular remodeling, particularly in patients with chronic kidney disease (CKD). However, metabolic determinants linking renal dysfunction to CAC remain incompletely understood. Ceramides, bioactive sphingolipids involved in vascular inflammation and calcification, may represent potential mediators of this interaction.

**Methods:**

In this cross-sectional analysis of 851 coronary artery disease (CAD) patients from the CRUISE-MET trial (NCT06383208), six plasma ceramide species [Cer(18:1/16:0), (18:1/18:0), (18:1/20:0), (18:1/22:0), (18:1/24:0), and (18:1/24:1)] were quantified by UHPLC–MS/MS. CAC severity was classified angiographically as absent, mild, moderate, or severe. Logistic regression, ROC analysis, and restricted cubic splines assessed associations. Joint and mediation analyses examined interactions with CKD. Sensitivity analyses evaluated consistency across subgroups and in a cohort with non-ECG-gated chest CT–derived CAC scores.

**Results:**

Patients with moderate-to-severe CAC had higher Cer(18:1/16:0), Cer(18:1/24:1), older age, higher SYNTAX scores, and lower estimated glomerular filtration rate (eGFR) (all p<0.05). Each SD increase in Cer(18:1/16:0) was associated with 59% higher odds of moderate-to-severe CAC (OR = 1.59, 95% CI: 1.33–1.88, p<0.001); the highest tertile was associated with 151% higher odds (OR = 2.51, 95% CI: 1.68–3.75, p<0.001). CKD modified this association (p for interaction=0.032), with coexisting CKD and high ceramide exposure yielding nearly fourfold higher odds (OR = 3.94, 95% CI: 2.47–6.28, p<0.001). Mediation analysis indicated Cer(18:1/16:0) accounted for 15.6% of the CKD–CAC association. Associations remained directionally consistent across sensitivity analyses. In the CT subgroup, modest associations were observed between selected ceramide species and quantitative CACS.

**Conclusion:**

Specific ceramides, particularly Cer(18:1/16:0), are independently associated with CAC severity, with stronger associations observed in patients with CKD.

## Highlights

- Higher plasma Cer(18:1/16:0) was independently associated with greater CAC severity.
- CKD modified the Cer(18:1/16:0)–CAC association, with combined CKD and high Cer(18:1/16:0) associated with the highest odds of moderate-to-severe CAC.
- Associations were directionally consistent in complementary CT-based analyses.

## Introduction

Coronary artery disease (CAD) remains the leading cause of death worldwide (1), and coronary artery calcification (CAC) represents a critical marker of advanced atherosclerosis (2) that increases the complexity of percutaneous coronary intervention (PCI) and predicts perioperative complications and long-term adverse outcomes (3–5). The burden of CAC is strongly age- and sex-dependent, exceeding 90% in men older than 70 years (3), and is amplified by diabetes mellitus (DM) (6), secondary hyperparathyroidism (7), and chronic kidney disease (CKD) (8). Among patients with CKD, CAC prevalence rises exponentially, from 60%–80% in stages 3–5 to more than 80% in dialysis populations (9), largely reflecting disordered calcium–phosphate metabolism (10). However, conventional mineral-targeted therapies, including phosphate binders and calcimimetics, have shown limited efficacy in slowing vascular calcification (11, 12), whereas statins may paradoxically accelerate CAC despite substantial LDL-cholesterol reduction (13). These observations highlight the need for novel biomarkers and therapeutic targets specific to the calcified coronary phenotype.

Lipid metabolic reprogramming is a shared pathophysiological feature of CKD (14) and a key modulator of vascular disease. Ceramides, central mediators in sphingolipid metabolism, exhibit significant nephrotoxicity (15, 16) that is independent of traditional cardio-renal risk factors (17). Patients with reduced renal function (eGFR <60 mL/min/1.73 m²) demonstrate elevated plasma levels of the ceramide species Cer(18:1/16:0) (18), which independently predicts acute kidney injury (AKI) risk in acute coronary syndrome (ACS) populations (19). On the other hand, sphingolipids exacerbate atherosclerosis (17) by promoting endothelial apoptosis, oxidized LDL uptake, and plaque instability. Consistent with this, prior studies have established strong links between elevated ceramide species to CAD severity (18, 20) and adverse cardiovascular outcomes (21, 22).

Arterial intimal calcification is considered a pathological hallmark of advanced atherosclerotic plaques and shares multiple upstream drivers with atherosclerosis, including CKD and systemic inflammation (2). Mechanistically, experimental studies have implicated ceramide signaling in the osteogenic transition of vascular smooth muscle cells (VSMCs) and vascular calcification, particularly under high-phosphate conditions (23). High phosphate rapidly increases ceramide accumulation in human VSMCs, whereas inhibition of acid sphingomyelinase (ASM) attenuates phosphate-induced osteogenic/chondrogenic differentiation and calcification through serum- and glucocorticoid-regulated kinase 1 (SGK1)-dependent signaling (23). In an atherosclerotic context, Ox-LDL-induced calcification of VSMCs has also been linked to Toll-like receptor 4 (TLR4)/nuclear factor-κB (NF-κB)/ceramide signaling (24) and neutral sphingomyelinase (N-SMase)/ceramide–p38 mitogen-activated protein kinase (p38 MAPK) signaling (25). However, these preclinical findings do not establish species-specific effects of the circulating ceramides evaluated in the present study. Within the CRUISE-MET registry, we have previously characterized the associations of circulating ceramides with coronary lesion complexity (26) and with coronary microvascular dysfunction (27). Whether specific plasma ceramide species relate to the calcified coronary phenotype, and whether renal dysfunction modifies this relationship, has not been established.

Accordingly, this study aimed to (1) quantify the associations of six ceramide species with angiographically graded CAC severity; (2) test whether renal function modifies these associations; and (3) explore potential pathways through joint and exploratory mediation analyses, with additional sensitivity analysis using non-ECG-gated chest CT-derived coronary artery calcium scores.

## Methods

### Study design and population

This cross-sectional analysis was embedded within the CRUISE-MET registry (NCT06383208) (28), a prospective cohort designed to characterize lipid metabolic reprogramming in patients with CAD and comorbid CKD for cardio-renal risk stratification; the registry design, screening procedures (2,121 patients screened; 965 enrolled between June 2022 and December 2024), and standardized biorepository protocols have been described previously (26, 27). For the present analysis, patients aged 18–80 years with angiography-confirmed CAD, documented renal function, and complete angiographic and ceramide data were eligible. We excluded patients with active malignancy requiring systemic therapy within 6 months, active infectious disease, systemic autoimmune disorders, Child-Pugh class C liver disease, or missing critical laboratory or angiographic data. After applying these criteria, 851 patients constituted the primary analytic cohort (**Supplementary Figure S1**). All participants provided written informed consent; the study was approved by the institutional ethics committee of China-Japan Friendship Hospital (approval No. 2024-KY-239) and was conducted in accordance with the Declaration of Helsinki.

### Data collection and definition

Demographic, clinical, and laboratory data were abstracted from electronic medical records by trained personnel into the REDCap platform (https://114.116.255.168/index.php). Recorded variables included age, sex, body mass index (BMI), systolic blood pressure (SBP), heart rate (HR), hypertension, diabetes mellitus (DM), smoking status, prior PCI, hemoglobin (Hb), fasting blood glucose (FBG), glycated hemoglobin (HbA1c), triglycerides (TG), total cholesterol (TC), low-density lipoprotein cholesterol (LDL-C), high-density lipoprotein cholesterol (HDL-C), serum calcium and phosphorus, urine albumin-to-creatinine ratio (UACR), and estimated glomerular filtration rate (eGFR). CKD was defined according to the 2024 KDIGO guideline as eGFR <60 mL/min/1.73 m² or UACR ≥30 mg/g with preserved eGFR (29), and eGFR was calculated using the 2021 CKD-EPI creatinine equation (30).

CAC severity was rigorously assessed by the independent core laboratory blinded to clinical and biomarker data. Based on angiographic findings, qualitative calcification grade was classified into four categories (3): (1) Absence; (2) Mild, faint opacities visible only in systole, resolving in diastole; (3) Moderate, distinct systolic opacities partially persisting; (4) Severe, persistent, clearly demarcated opacities regardless of motion. The baseline SYNTAX score (bSS) was calculated using a validated web-based calculator (https://syntaxscore.org/). A bSS >22 was classified as complex CAD (31). Angiograms were acquired using standardized techniques (≥15 frames/sec, isocentric imaging) and evaluated by dual-certified interventional cardiologists, with discrepancies resolved by third-party adjudication (31).

### Blood sample collection and quantification of ceramides

Fasting venous blood samples (4 mL) were collected in EDTA tubes, centrifuged within 2 h, and plasma aliquots were stored at −80 °C until analysis. Plasma ceramide profiling was performed using a validated UHPLC–MS/MS platform (AB Sciex Qtrap 6500+ coupled with Waters I-class UPLC) with isotope-labeled internal standards for quality control and reproducibility, as previously described (32). Six ceramide species were quantified: Cer(18:1/16:0), Cer(18:1/18:0), Cer(18:1/20:0), Cer(18:1/22:0), Cer(18:1/24:0), and Cer(18:1/24:1). Absolute concentrations were determined using external calibration combined with internal standardization and expressed as ng/mL (33–35). Quality control samples were analyzed throughout the batch, with >80% of detected metabolites showing coefficients of variation <20%, indicating satisfactory analytical stability.

### Statistical analysis

Continuous variables were summarized as means ± standard deviations (SD) for normally distributed data or medians with interquartile ranges (IQR) for skewed distributions. Intergroup comparisons across three severity strata were performed using ANOVA (parametric) or Kruskal-Wallis tests (nonparametric). Differences in plasma ceramide species between groups were visualized using boxplots. Categorical variables are expressed as frequencies (percentages) and analyzed using χ² tests. To address missing data, multiple imputation via fully conditional specification was performed, preserving analytical validity. Specific ceramide species were log-transformed prior to analysis when necessary. All statistical analyses were performed using the R Programming Language 4.4.1 (Vienna, Austria), MedCalc 19.1 (Ostend, Belgium) and Zstats 1.0 (www.zstats.net), with statistical significance defined as two-tailed *p* < 0.05.

Associations among plasma ceramide species, angiographic CAC grade, and other clinical parameters were assessed using Spearman rank correlation analysis, with results visualized in a correlation heatmap. Logistic regression evaluated associations between plasma ceramide species and the risk of moderate-to-severe CAC, adjusting for confounders. Variables with potential significance identified by univariate logistic regression analysis (p < 0.1) and those clinically relevant to CAC were included in the multivariable logistic regression model. Three sequential models were constructed: Model I (unadjusted) assessed crude associations; Model II adjusted for sex, age, BMI, previous PCI, hypertension, DM, and smoking status; Model III further adjusted for CKD, TG, LDL-C, HDL-C, Ca²^+^, P, and antiplatelet/statin use. Ceramide concentrations were analyzed as both continuous variables (per SD increase) and categorical variables (tertiles: T1–T3). Trend tests across tertiles were performed by treating the tertile categories as an ordinal variable. Restricted cubic spline (RCS) models were applied to examine dose-response relationships between plasma ceramide levels (continuous) and moderate-to-severe CAC, adjusting for multivariable confounders included in Model III.

To assess the robustness of our findings, we conducted extensive sensitivity analyses. First, we utilized an artificial intelligence–based tool to compute quantitative Agatston scores from non-electrocardiogram-gated chest CT scans using a commercially available software (uAI Discover^®^ Nongated CACS, United Imaging Intelligence, Shanghai, China) (36–38) in a predefined subgroup of the cohort, after excluding patients with prior PCI and those without available chest CT imaging. In this subgroup, the association between plasma ceramide levels and calcification burden was evaluated using linear regression models. Detailed methodology for image analysis is provided in the **Supplementary Methods**. Furthermore, we performed additional sensitivity analyses in four prespecified clinical subgroups: (1) the complete renal biomarker cohort, after excluding patients with incomplete UACR measurements (14.92%); (2) patients with a history of statin therapy; (3) patients with DM; and (4) patients with comorbid hypertension. Finally, we performed predefined subgroup analyses stratified by key clinical variables, including age (<65 vs. ≥65 years), sex, BMI (<24 vs. ≥24 kg/m²), previous PCI, hypertension, DM, smoking status, prior statin use, and CKD. The associations between ceramide levels (per SD) and moderate-to-severe CAC were evaluated across all subgroups. Interaction effects were formally tested using likelihood ratio tests, comparing models with and without interaction terms.

Receiver operating characteristic (ROC) curve analysis evaluated the discriminative ability of individual ceramide species for moderate-to-severe CAC, with comparisons by DeLong’s test. Plasma ceramide levels were stratified by optimal cut-off values and combined with CKD status (non-CKD vs. CKD) to assess joint effects. Additive interaction between CKD and elevated Cer(18:1/16:0) was assessed using the relative excess risk due to interaction (RERI), attributable proportion due to interaction (AP), and synergy index (S). These measures were estimated using modified Poisson regression with robust standard errors, using the same covariate sets as Models I–III. Exploratory mediation analysis was performed to examine statistical indirect associations among CKD, Cer(18:1/16:0), and CAC (R package *mediation*, v4.2.2). Bayesian methods estimated direct and indirect effects, adjusting for sex, age, BMI, prior PCI, hypertension, DM, smoking, TG, LDL-C, HDL-C, Ca²^+^, P, and antiplatelet/statin use. The reciprocal mediating effect of ceramide on the CKD–CAC association was also assessed.

## Results

### Baseline characteristics

The final analytic cohort comprised 851 eligible patients (mean age 64 ± 10 years). Baseline characteristics stratified by CAC severity are presented in **Table 1**. Compared to patients with none or mild CAC, those with moderate-to-severe CAC (n=364, 42.77%) were significantly older and exhibited higher levels of serum phosphorus, UACR, bSS, Cer (18:1/16:0), and Cer (18:1/24:1), alongside lower BMI, Hb, left ventricular ejection fraction (LVEF), and eGFR (all p < 0.05). This group also had a significantly higher prevalence of DM, CKD, complex CAD, and antidiabetic medication use (*p* < 0.05). In contrast, no significant differences were observed between the groups in sex distribution, history of hypertension, smoking status, previous PCI, SBP, HR, FBG, glycated hemoglobin (HbA1c), lipid profiles (TG, TC, LDL-C, HDL-C), serum calcium, concentrations of other measured ceramide species [Cer(18:1/18:0), Cer(18:1/20:0), Cer(18:1/22:0), Cer(18:1/24:0)], or use of cardiovascular medications (including antiplatelet agents, statins, ezetimibe, or PCSK9 inhibitors). Additionally, differences in ceramide concentrations between CAC severity groups are visually presented in **Figure 1**. When CAC was retained as four angiographic categories (absent, mild, moderate, and severe), Cer(18:1/16:0) showed an overall increase with greater calcification severity, whereas differences between the moderate and severe categories were less apparent for several other ceramide species (**Supplementary Table S1**).

**Table 1:** Baseline characteristics stratified by severity of coronary calcification.

Variables	None (n = 265)	Mild (n = 222)	Moderate/severe (n = 364)	*p*
Age, years	60 ± 11	64 ± 10	66 ± 9	<.001
Female, n (%)	64 (24.15)	58 (26.13)	107 (29.40)	0.327
BMI, kg/m^2^	26.69 ± 4.62	26.42 ± 3.99	25.61 ± 3.31	0.002
Hypertension, n (%)	201 (75.85)	184 (82.88)	300 (82.42)	0.070
DM, n (%)	147 (55.47)	132 (59.46)	253 (69.51)	<.001
Smoking, n (%)	123 (46.42)	93 (41.89)	155 (43.18)	0.571
Previous PCI, n (%)	92 (34.72)	63 (28.38)	109 (29.95)	0.271
SBP, mmHg	134 ± 21	136 ± 23	136 ± 21	0.777
HR, bpm	77 ± 14	79 ± 15	78 ± 14	0.359
Hb, g/L	139 ± 22	136 ± 22	129 ± 23	<.001
FBG, mmol/L	8.83 ± 4.50	9.22 ± 6.15	9.00 ± 4.20	0.683
HbA1c, %	6.88 ± 1.42	7.04 ± 1.58	7.12 ± 1.49	0.147
TG, mmol/L	1.59 (1.15,2.21)	1.66 (1.13,2.35)	1.51 (1.06,2.29)	0.531
TC, mmol/L	3.93 (3.19,4.92)	3.93 (3.15,5.03)	3.88 (3.10,4.91)	0.642
LDL-C, mmol/L	2.27 (1.80,3.07)	2.37 (1.84,3.15)	2.25 (1.76,3.04)	0.448
HDL-C, mmol/L	1.06 (0.90,1.22)	1.06 (0.91,1.25)	1.10 (0.91,1.30)	0.161
Ca^2+^, mmol/L	2.29 ± 0.13	2.30 ± 0.14	2.30 ± 0.15	0.924
P, mmol/L	1.11 ± 0.23	1.15 ± 0.25	1.21 ± 0.33	<.001
UACR, mg/g	6.14 (0.17,25.97)	9.03 (0.59,47.52)	11.51 (0.38,73.23)	0.010
eGFR, mL/min/1.73m^2^	91.37 (76.70,104.45)	86.35 (57.84,99.12)	76.27 (43.80,94.26)	<.001
CKD, n (%)	81 (30.57)	97 (43.69)	188 (51.65)	<.001
bSS	12 (7,18)	17 (10,27)	26 (16,37)	<.001
Complex CAD	38 (14.34)	84 (37.84)	222 (60.99)	<.001
LVEF, %	63 ± 9	60 ± 12	62 ± 10	0.027
CAD subtype				0.065
CCS	67 (25.28)	80 (36.04)	111 (30.49)	
UA	109 (41.13)	71 (31.98)	122 (33.52)	
AMI	89 (33.58)	71 (31.98)	131 (35.99)	
Cer (18:1/16:0), ng/mL	338.99 (274.30,430.17)	355.14 (275.31,451.29)	391.42 (295.07,510.64)	<.001
Cer (18:1/18:0), ng/mL	81.56 (56.56,106.43)	79.85 (52.78,108.10)	85.44 (58.45,125.89)	0.054
Cer (18:1/20:0), ng/mL	51.80 (37.10,73.10)	53.46 (35.44,71.51)	54.84 (35.51,83.25)	0.405
Cer (18:1/22:0), ng/mL	5880.87 (4386.54,8264.81)	6108.61 (4334.34,8073.92)	5908.52 (4359.97,8603.27)	0.954
Cer (18:1/24:0), ng/mL	1348.87 (974.47,1748.46)	1368.65 (948.53,1851.00)	1299.05 (960.87,1815.10)	0.902
Cer (18:1/24:1), ng/mL	649.34 (471.45,862.52)	664.27 (472.51,825.44)	721.36 (521.14,940.10)	0.007
Medication history				
Antiplatelet drugs, n (%)	175 (66.04)	139 (62.61)	244 (67.03)	0.540
Statins, n (%)	168 (63.40)	140 (63.06)	211 (57.97)	0.295
Ezetimibe, n (%)	77 (29.06)	64 (28.83)	101 (27.75)	0.927
PCSK-9i, n (%)	40 (15.09)	23 (10.36)	49 (13.46)	0.298
Antidiabetic agents, n (%)	132 (49.81)	110 (49.55)	222 (61.84)	0.002

BMI, body mass index; PCI, percutaneous coronary intervention; SBP, systolic blood pressure; HR, heart rate; FBG, fasting blood glucose; TG, triglyceride; TC, total cholesterol; HDL-C, high density lipoprotein cholesterol; LDL-C, low density lipoprotein cholesterol; UACR, urine albumin-to-creatinine ratio; eGFR, estimating glomerular filtration rate; CKD, chronic kidney disease; Ca^2+^, serum calcium; P, serum phosphorus; LVEF, left ventricular ejection fraction; Cer, Ceramide; bSS, baseline SYNTAX score; PCSK-9i, proprotein convertase subtilisin/kexin type 9 inhibitors.

**Figure 1:**
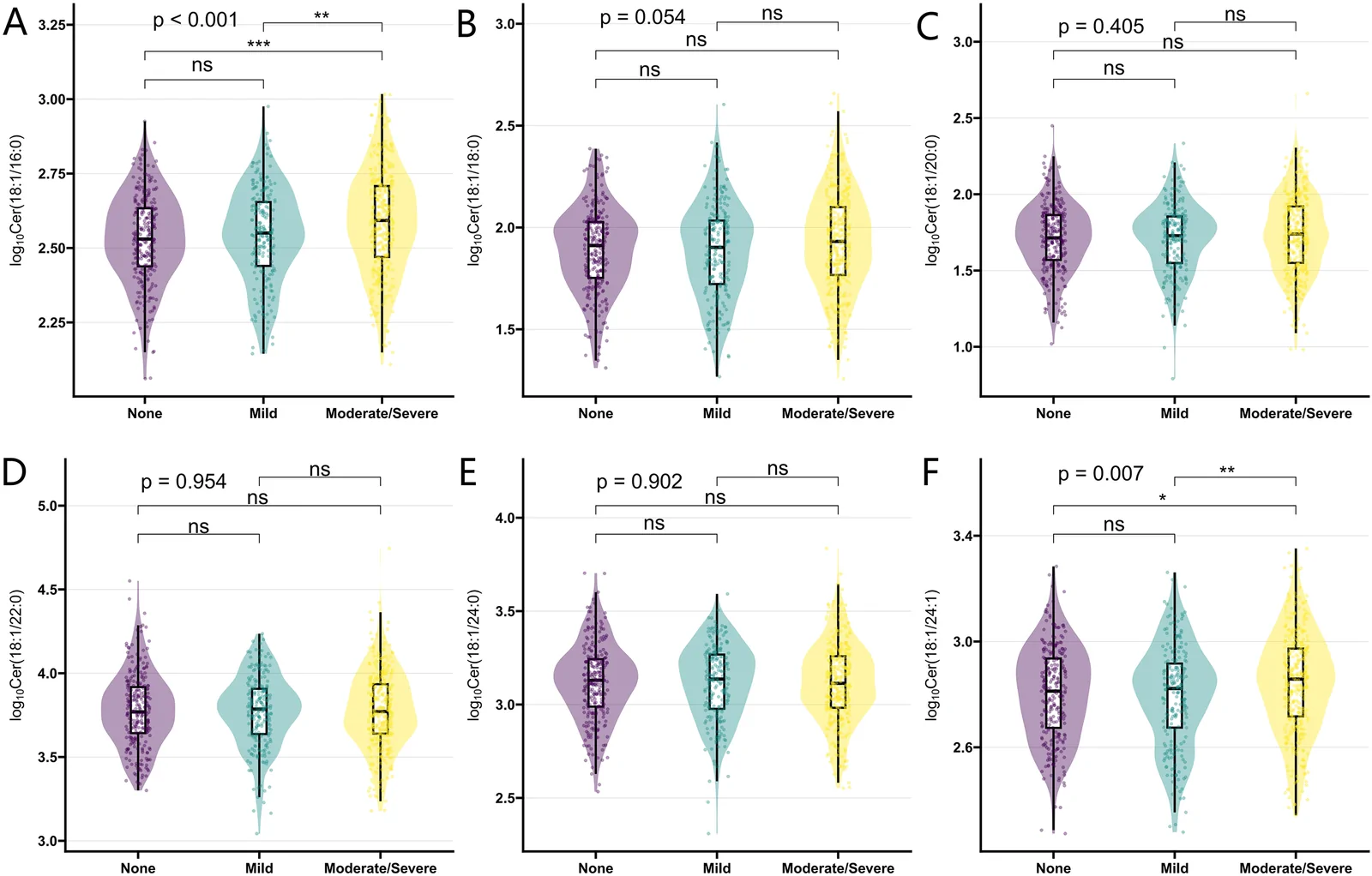
Plasma ceramide concentrations across CAC severity groups. Log-transformed plasma concentrations of six ceramide species across CAC severity groups **(A–F)**. Significance codes: **p < 0.001; *p < 0.01; *p* < 0.05; ns, *p* ≥ 0.05. CAC, coronary artery calcification; Cer, ceramide. *** indicates p < 0.001.

### Association of plasma ceramide with coronary calcification

Spearman rank correlation analysis (**Supplementary Figure S2**) demonstrated significant positive correlations of CAC grade with Cer(18:1/16:0) (ρ = 0.157, p < 0.001), Cer(18:1/18:0) (ρ = 0.072, p = 0.035), and Cer(18:1/24:1) (ρ = 0.081, p = 0.019). CAC grade was also negatively correlated with eGFR and BMI and positively correlated with serum phosphorus, bSS, and age.

Logistic regression analyses (**Table 2**; **Supplementary Tables 2, S3**) revealed distinct associations between specific plasma ceramide species and the risk of moderate-to-severe CAC. Notably, Ceramides Cer(18:1/16:0), Cer(18:1/18:0), and Cer(18:1/24:1) demonstrated significant positive associations in both continuous (per SD increase) and categorical (tertile) analyses. For Cer (18:1/16:0), each standard deviation (SD) increase in plasma concentration was associated with a 59% higher odds of moderate-to-severe CAC (OR = 1.59, 95% CI: 1.33–1.88, *p* < 0.001) in the fully adjusted model (Model III). Tertile-based analysis further revealed a dose-response pattern: participants in the highest tertile (T3) had 2.51-fold higher odds of moderate-to-severe CAC compared to the lowest tertile (T1) (OR = 2.51, 95% CI: 1.68–3.75, *p* < 0.001), with a significant linear trend across tertiles (*p* for trend < 0.001). Consistent positive associations were observed for Cer(18:1/16:0) in unadjusted (Model I) and minimally adjusted models (Model II). Similar significant positive associations were observed for Cer(18:1/18:0) and Cer(18:1/24:1) across all models. In contrast, Cer(18:1/20:0), Cer(18:1/22:0), and Cer(18:1/24:0) showed no statistically significant associations with moderate-to-severe CAC risk in adjusted analyses (all *p* > 0.05).

**Table 2:** Logistic regression analysis of plasma ceramide concentrations and coronary calcification.

	Model I		Model II		Model III	
	OR (95% CI)	*p*	OR (95% CI)	*p*	OR (95% CI)	*p*
Cer (18:1/16:0) (per SD)	1.46 (1.27 ~ 1.68)	<.001	1.57 (1.34 ~ 1.82)	<.001	1.59 (1.33 ~ 1.88)	<.001
Cer (18:1/16:0) (tertiles)						
T1	1.00 (Reference)		1.00 (Reference)		1.00 (Reference)	
T2	1.16 (0.83 ~ 1.63)	0.386	1.16 (0.81 ~ 1.66)	0.416	1.15 (0.80 ~ 1.67)	0.449
T3	2.10 (1.50 ~ 2.95)	<.001	2.48 (1.72 ~ 3.57)	<.001	2.51 (1.68 ~ 3.75)	<.001
*p* value for trend	<.001		<.001		<.001	
Cer (18:1/18:0) (per SD)	1.25 (1.09 ~ 1.43)	0.002	1.42 (1.22 ~ 1.65)	<.001	1.41 (1.20 ~ 1.66)	<.001
Cer (18:1/18:0) (tertiles)						
T1	1.00 (Reference)		1.00 (Reference)		1.00 (Reference)	
T2	1.07 (0.77 ~ 1.50)	0.684	1.12 (0.79 ~ 1.59)	0.520	1.13 (0.78 ~ 1.62)	0.520
T3	1.40 (1.00 ~ 1.95)	0.051	1.68 (1.17 ~ 2.41)	0.005	1.62 (1.10 ~ 2.37)	0.014
*p* value for trend	0.043		0.004		0.012	
Cer (18:1/24:1) (per SD)	1.28 (1.12 ~ 1.47)	<.001	1.40 (1.21 ~ 1.62)	<.001	1.39 (1.18 ~ 1.64)	<.001
Cer (18:1/24:1) (tertiles)						
T1	1.00 (Reference)		1.00 (Reference)		1.00 (Reference)	
T2	1.17 (0.84 ~ 1.64)	0.354	1.19 (0.84 ~ 1.69)	0.327	1.16 (0.81 ~ 1.66)	0.427
T3	1.59 (1.14 ~ 2.23)	0.007	1.87 (1.31 ~ 2.68)	<.001	1.76 (1.19 ~ 2.61)	0.005
*p* value for trend	0.006		<.001		0.004	

Model I: Crude.

Model II: Adjusted for sex, age, BMI, previous PCI, hypertension, diabetes mellitus, smoking.

Model III: Adjusted for sex, age, BMI, previous PCI, hypertension, diabetes mellitus, smoking, CKD, TG, LDL-C, HDL-C, Ca2+, P, antiplatelet agents use and statins use.

BMI, body mass index; TG, triglyceride; HDL-C, high density lipoprotein cholesterol; LDL-C, low density lipoprotein cholesterol; CKD, chronic kidney disease; Ca^2+^, serum calcium; P, serum phosphorus; Cer, ceramide; CI, confidence interval; OR, odds ratio; SD, standard deviation.

Restricted cubic spline analyses confirmed linear dose-response relationships for these ceramides (**Figure 2**). Cer (18:1/16:0) demonstrated a pronounced positive association with calcification risk (overall *p* < 0.001; nonlinear *p* = 0.077), with risk proportionally rising as concentrations increased. Parallel trends were observed for Cer (18:1/18:0) (overall *p* = 0.001; nonlinear *p* = 0.577) and Cer (18:1/24:1) (overall *p* = 0.002; nonlinear *p* = 0.647), underscoring their dose-dependent effects.

**Figure 2:**
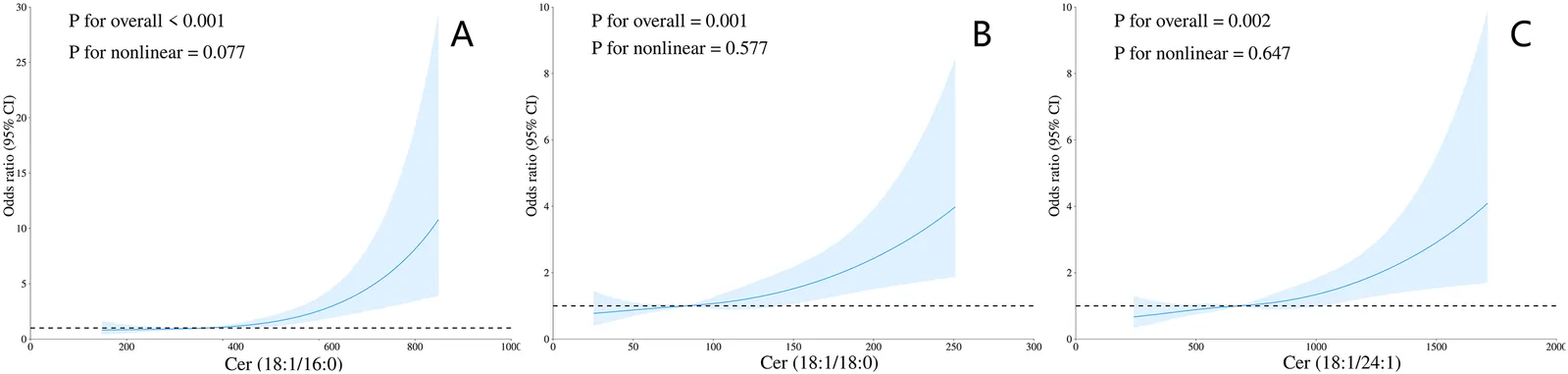
Dose-response relationships between plasma ceramide concentrations and CAC. Dose-response relationships between plasma ceramide species and CAC **(A–C)**, adjusted for sex, age, previous PCI, HTN, DM, smoking, CKD, BMI, TG, LDL-C, HDL-C, Ca²^+^, P, antiplatelet use, and statin use. CAC, coronary artery calcification; PCI, percutaneous coronary intervention; HTN, hypertension; DM, diabetes mellitus; CKD, chronic kidney disease; BMI, body mass index; TG, triglyceride; LDL-C, low-density lipoprotein cholesterol; HDL-C, high-density lipoprotein cholesterol; Ca²^+^, serum calcium; P, serum phosphorus.

### Sensitivity analysis

In a sensitivity analysis limited to participants with non-electrocardiogram-gated chest CT scans (n = 280), baseline characteristics according to CACS categories are summarized in **Supplementary Table S4**. Scatter plots (**Figure 3**) demonstrated weak but statistically significant positive correlations between plasma ceramide concentrations and CACS for log-transformed Cer(18:1/16:0) (r = 0.219, p < 0.001), Cer(18:1/18:0) (r = 0.159, p = 0.008), Cer(18:1/20:0) (r = 0.132, p = 0.027), and Cer(18:1/24:1) (r = 0.153, p = 0.010). In multivariable-adjusted linear regression analyses, Cer(18:1/16:0) and Cer(18:1/18:0) remained significantly associated with log-transformed CACS, with significant trends across increasing ceramide levels (all p for trend < 0.05), after adjustment for conventional cardiovascular risk factors (**Supplementary Table S5**). These findings provide complementary quantitative support for the associations observed in the primary angiographic analyses, although the magnitudes of the unadjusted correlations were modest. The associations between ceramide species and the risk of moderate-to-severe CAC remained consistent across other validation cohorts (**Supplementary Tables S6–S9**): (1) the complete renal biomarker cohort (n = 724), (2) patients with a history of statin therapy (n = 519), (3) patients with DM (n = 532), and (4) patients with comorbid hypertension (n = 685).

**Figure 3:**
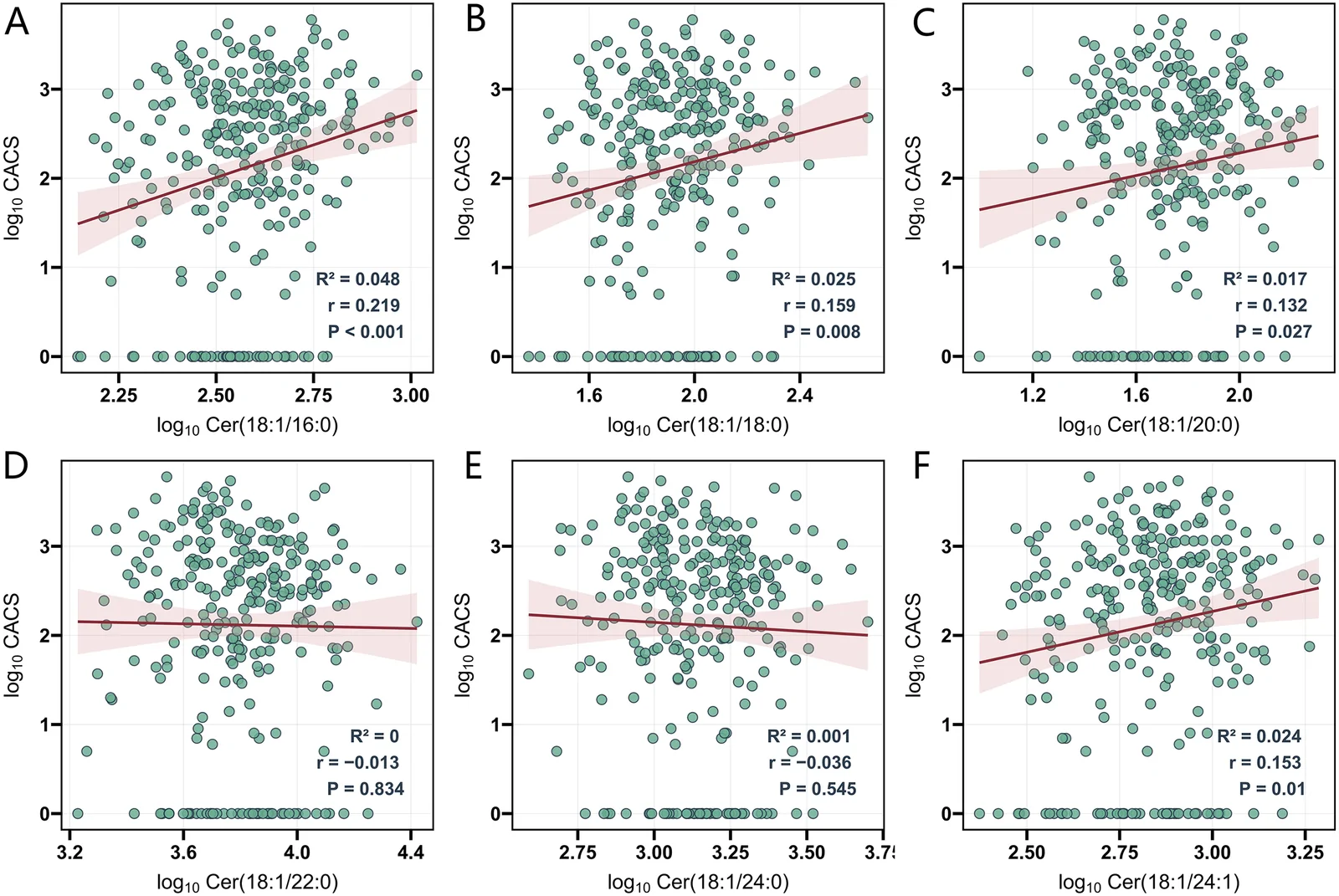
Associations between plasma ceramide concentrations and coronary artery calcium score in participants undergoing non-electrocardiogram-gated chest CT. **(A–F)** Show scatter plots with regression lines for the associations between log-transformed plasma ceramide concentrations and log-transformed CACS. Cer, ceramide; CACS, coronary artery calcium score; CT, computed tomography; *r*, Pearson correlation coefficient; R², coefficient of determination.

Subgroup analyses (**Figure 4**) showed consistent associations between per-SD increases in Cer(18:1/16:0) and moderate-to-severe CAC across all prespecified subgroups (all p < 0.05). A significant interaction with CKD status (p = 0.032) indicated stronger associations in CKD patients (OR = 1.88, 95% CI: 1.45–2.46) than in non-CKD individuals (OR = 1.44, 95% CI: 1.12–1.86). No significant interactions were found for age category, sex, BMI category, previous PCI, hypertension, DM, smoking, or statin use (all p for interaction > 0.05). A similar CKD-dependent interaction occurred for Cer(18:1/24:1) (p = 0.035). Detailed subgroup results for Cer(18:1/18:0) and Cer(18:1/24:1) are shown in **Supplementary Figures S3, S4**.

**Figure 4:**
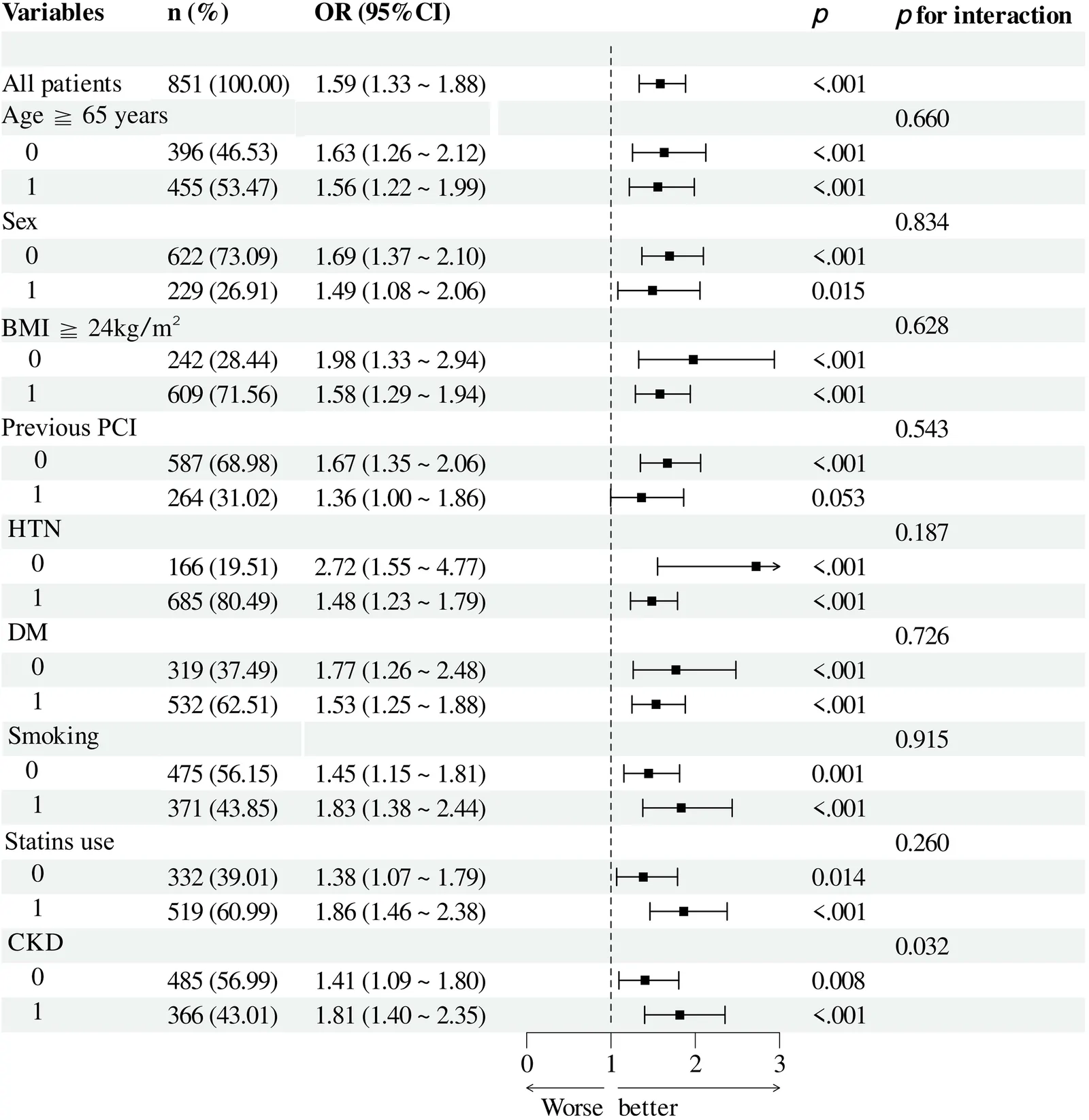
Subgroup analyses of the association between Cer(18:1/16:0) and CAC. Adjusted ORs and 95% CIs per SD increase in Cer(18:1/16:0) are shown. Models were adjusted for sex, age, previous PCI, HTN, DM, smoking, CKD, BMI, TG, LDL-C, HDL-C, Ca²^+^, P, antiplatelet use, and statin use. Cer, ceramide; OR, odds ratio; CI, confidence interval; SD, standard deviation; PCI, percutaneous coronary intervention; HTN, hypertension; DM, diabetes mellitus; CKD, chronic kidney disease; BMI, body mass index; TG, triglyceride; LDL-C, low-density lipoprotein cholesterol; HDL-C, high-density lipoprotein cholesterol; Ca²^+^, serum calcium; P, serum phosphorus.

### Joint association and exploratory mediation analyses of plasma Cer (18:1/16:0) and CKD on CAC

ROC analysis showed limited discrimination of plasma ceramide species for moderate-to-severe CAC. Cer(18:1/16:0) demonstrated the highest discriminative ability among all species, with an AUC of 0.589 (95%CI: 0.550–0.628; *p* < 0.001) and an optimal cutoff >432.48 ng/mL (**Supplementary Figure S5**). Patients stratified by CKD status and Cer(18:1/16:0) levels (dichotomized at this cutoff) exhibited distinct risk profiles. Using the Non-CKD/Low-Cer(18:1/16:0) group as the reference, fully adjusted models showed that the CKD/High-Cer(18:1/16:0) group had the highest odds of moderate-to-severe CAC (OR = 3.94, 95% CI: 2.46–6.30; *p* < 0.001; **Table 3**). Formal additive interaction analyses yielded positive point estimates; however, the associations were attenuated after multivariable adjustment. In Model III, the RERI was 0.36 (95% CI: −0.04 to 0.76), the AP was 0.20 (95% CI: −0.02 to 0.41), and the synergy index was 1.76 (95% CI: 0.77–4.04), with all confidence intervals including their corresponding null values (**Table 3**). Thus, the fully adjusted analysis did not provide statistically significant evidence of additive interaction.

**Table 3:** Joint association and additive interaction of Cer(18:1/16:0) and CKD with moderate-to-severe CAC.

Panel A. Joint association of CKD and Cer(18:1/16:0) with moderate-to-severe CAC						
	Model I		Model II		Model III	
	OR (95% CI)	*p*	OR (95% CI)	*p*	OR (95% CI)	*p*
Non-CKD/ L-Cer (18:1/16:0)	1.00 (Reference)		1.00 (Reference)		1.00 (Reference)	
CKD/L-Cer (18:1/16:0)	1.37 (0.97 ~ 1.93)	0.075	1.23 (0.86 ~ 1.77)	0.252	1.21 (0.84 ~ 1.76)	0.307
Non-CKD/ H-Cer (18:1/16:0)	1.31 (0.88 ~ 1.97)	0.188	1.59 (1.03 ~ 2.45)	0.035	1.71 (1.08 ~ 2.71)	0.021
CKD/H-Cer (18:1/16:0)	3.84 (2.53 ~ 5.83)	<.001	3.92 (2.54 ~ 6.06)	<.001	3.94 (2.46 ~ 6.30)	<.001
*p* value for trend	<.001		<.001		<.001	
Panel B. Additive interaction between CKD and elevated Cer(18:1/16:0)						
Interaction measure	Model I	*p*	Model II	*p*	Model III	*p*
RERI (95% CI)	0.54 (0.16–0.93)	0.006	0.43 (0.03–0.82)	0.034	0.36 (−0.04–0.76)	0.079
AP (95% CI)	0.28 (0.09–0.47)	0.004	0.23 (0.02–0.43)	0.029	0.20 (−0.02–0.41)	0.070
S (95% CI)	2.36 (0.88–6.28)	0.087	1.98 (0.81–4.86)	0.135	1.76 (0.77–4.04)	0.182

Model I: Crude.

Model II: Adjusted for sex, age, BMI, previous PCI, HTN, DM, and smoking.

Model III: Further adjusted for TG, LDL-C, HDL-C, Ca²^+^, P, antiplatelet use, and statin use.

Panel A presents ORs from logistic regression. Panel B presents additive interaction measures estimated using modified Poisson regression with robust standard errors. RERI > 0, AP > 0, and S > 1 indicate positive additive interaction.

L-Cer, Cer(18:1/16:0) ≤432.48 ng/mL; H-Cer, Cer(18:1/16:0) >432.48 ng/mL.

AP, attributable proportion due to interaction; BMI, body mass index; Cer, ceramide; CI, confidence interval; CKD, chronic kidney disease; DM, diabetes mellitus; HTN, hypertension; HDL-C, high-density lipoprotein cholesterol; LDL-C, low-density lipoprotein cholesterol; OR, odds ratio; PCI, percutaneous coronary intervention; RERI, relative excess risk due to interaction; S, synergy index; TG, triglyceride; Ca²^+^, serum calcium; P, serum phosphorus.

Given the observed joint associations between CKD, Cer(18:1/16:0), and CAC, exploratory mediation analyses were subsequently performed to characterize their statistical indirect relationships. Elevated Cer(18:1/16:0) accounted for 15.63% of the statistical indirect association between CKD and CAC severity (**Figure 5D**), whereas CKD accounted for 4.04% of the statistical indirect association between Cer(18:1/16:0) and CAC in the fully adjusted model (**Figure 5B**).

**Figure 5:**
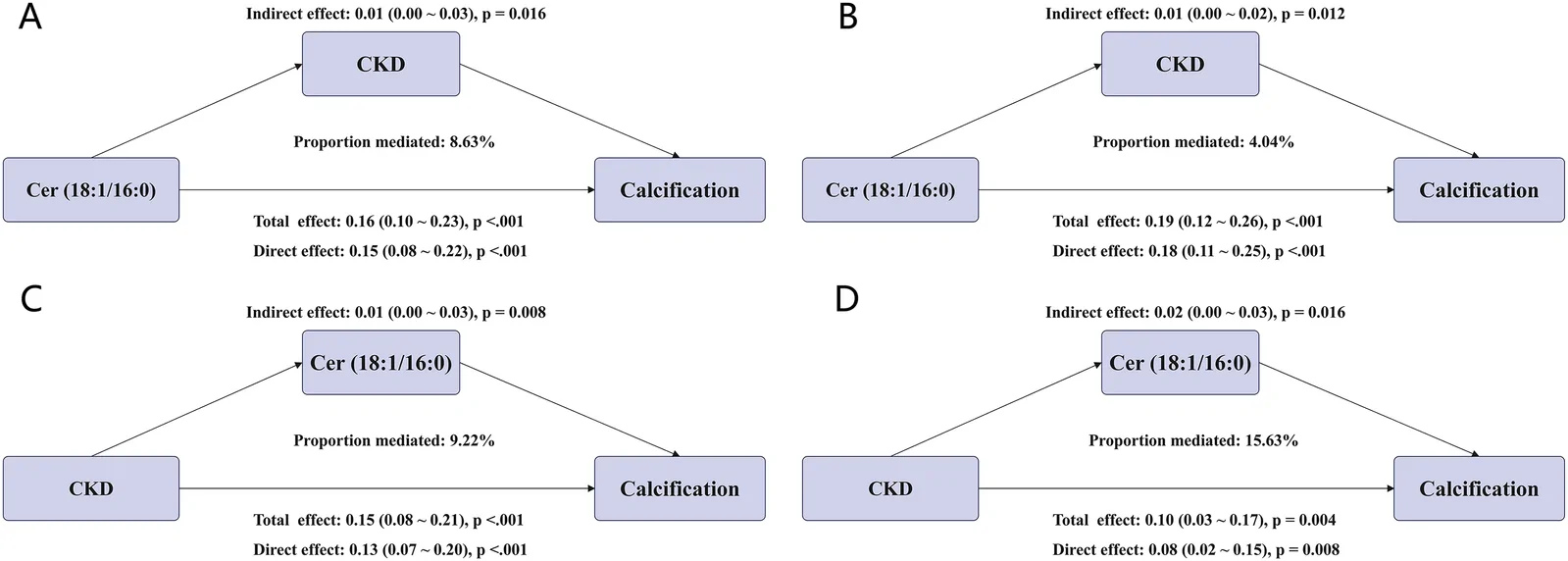
Exploratory mediation analyses of the associations among Cer(18:1/16:0), CKD, and CAC. **(A, C)** Show unadjusted analyses, whereas **(B, D)** show analyses adjusted for sex, age, previous PCI, HTN, DM, smoking, BMI, TG, LDL-C, HDL-C, Ca²^+^, P, antiplatelet use, and statin use. Cer, ceramide; CKD, chronic kidney disease; PCI, percutaneous coronary intervention; HTN, hypertension; DM, diabetes mellitus; BMI, body mass index; TG, triglyceride; LDL-C, low-density lipoprotein cholesterol; HDL-C, high-density lipoprotein cholesterol; Ca²^+^, serum calcium; P, serum phosphorus.

## Discussion

This cross-sectional analysis of the CRUISE-MET cohort identified three principal findings: (1) Specific ceramide species (Cer(18:1/16:0), Cer(18:1/24:1)) demonstrate dose-dependent associations with angiographic CAC severity (p for trend < 0.001), independent of traditional risk factors. Consistent results were observed across multiple sensitivity analyses; (2) CKD modified the association between Cer(18:1/16:0) and CAC (*p* for interaction = 0.032), with the combined presence of CKD and high Cer(18:1/16:0) associated with the highest odds of CAC; (3) exploratory mediation analyses suggested statistical indirect associations among CKD, Cer(18:1/16:0), and CAC, although temporal and causal relationships cannot be established from the present cross-sectional data. Collectively, these findings support an association between specific circulating ceramides and the calcified coronary phenotype, particularly in the setting of renal dysfunction.

These findings extend our previous CRUISE-MET analysis, in which circulating ceramides and renal dysfunction were associated with coronary lesion complexity assessed by the SYNTAX score (26). The present study focuses on a distinct coronary phenotype—CAC. Whereas the SYNTAX score reflects the overall anatomical extent and complexity of CAD, CAC represents a mineralized vascular remodeling phenotype closely linked to osteogenic transition of VSMCs and CKD-associated mineral stress. The consistent association of Cer(18:1/16:0) across these different coronary phenotypes supports a broader relationship between ceramide remodeling and CKD-associated coronary vascular disease, while the present findings specifically extend this relationship to coronary calcification.

The association between circulating ceramides and CAC remained consistent across multiple sensitivity analyses and after adjustment for traditional risk factors such as age (39), DM (40), CKD (9), and prior statin therapy (41, 42). Although statin exposure may influence the CAC phenotype (41), the association between ceramide levels and CAC remained consistent after adjustment for statin use and in the statin-treated sensitivity analysis. While the underlying mechanisms remain incompletely understood, the observed association is biologically plausible and warrants further investigation. Atherosclerosis and intimal vascular calcification are closely intertwined pathological processes, with inflammation, lipid deposition, and oxidative stress contributing to the osteogenic transformation of VSMCs (2, 43–45). Ceramides are bioactive sphingolipids capable of amplifying inflammatory, oxidative, and pro-apoptotic signaling, providing a plausible link between altered sphingolipid metabolism and a vascular microenvironment permissive for calcification (17, 44, 45). Thus, ceramide dysregulation may represent one of several metabolic pathways connecting advanced atherosclerosis with calcific remodeling.

The potential role of ceramide in vascular calcification builds upon its well-established contributions to atherosclerosis pathogenesis (17, 18). Ceramide signaling can promote endothelial dysfunction, oxidative stress, inflammatory activation, and lipid accumulation (17, 46, 47). Our previous work further identified CysLT2R and P2RY6 as ceramide-responsive receptors whose activation engages Gq-dependent signaling and downstream inflammasome pathways (18). More directly, experimental studies have linked sphingomyelinase–ceramide signaling to osteogenic transition of VSMCs. In human VSMCs, Ox-LDL-induced calcification involved TLR4/NF-κB/ceramide signaling, TLR4 knockdown reduced calcium deposition and osteogenic marker expression, while NF-κB inhibition attenuated calcification, an effect partially rescued by C2-ceramide (24). Complementary experiments further showed that Ox-LDL activates N-SMase/ceramide signaling and downstream p38 MAPK, promoting osteogenic differentiation, alkaline phosphatase activity, and mineralization of VSMCs (25). Similarly, acid sphingomyelinase (ASM)/ceramide signaling promoted osteo-/chondrogenic transdifferentiation of VSMCs and calcification through SGK1-dependent pathways, particularly under high-phosphate conditions (23). Under high-phosphate exposure, ceramide levels increased rapidly, whereas ASM inhibition or deficiency attenuated osteogenic/chondrogenic differentiation, alkaline phosphatase activity, and calcification (23). These findings provide experimental support for a sphingomyelinase–ceramide signaling axis in VSMCs, although they do not establish a species-specific causal role for the circulating ceramides measured in the present study.

Another significant finding is that CKD status substantially modifies the association between ceramides and coronary calcification. Patients with coexisting CKD and elevated Cer(18:1/16:0) had the highest odds of moderate-to-severe CAC. Disturbances in calcium-phosphate metabolism are major drivers of vascular calcification in CKD (10), yet currently available mineral-targeted therapies have shown limited efficacy in attenuating its progression (11, 12). Experimental evidence provides a potential mechanistic point of convergence between mineral stress and sphingolipid signaling: high-phosphate conditions increase ceramide accumulation in VSMCs, while ASM/ceramide signaling enhances phosphate-induced osteogenic transdifferentiation through SGK1-dependent pathways (23).

Previous studies have established that ceramides not only contribute to atherosclerosis through inflammatory pathways but are also closely associated with renal impairment (16). Ceramide-driven renal injury and fibrotic remodeling are orchestrated through pleiotropic mechanisms, including: induction of apoptosis, disruption of insulin signaling, impairment of mitochondrial fatty acid β-oxidation, and potentiation of oxidative stress (16, 48). Together, these findings suggest that CKD-associated mineral and metabolic disturbances may provide a biological context in which ceramide-related signaling becomes more relevant to vascular calcification. However, the present observational data cannot determine whether these mechanisms explain the stronger ceramide–CAC association observed in patients with CKD.

These findings support the potential value of ceramide profiling for characterizing the metabolic phenotype associated with CAC, although its incremental clinical utility requires further evaluation. Furthermore, the observed associations raise the possibility that ceramide signaling may be involved in vascular calcification in the setting of CKD; however, the present findings do not establish that targeting this pathway would modify calcification burden. Clinically validated lipid-lowering approaches, including lifestyle interventions (e.g., Mediterranean diet (49), regular aerobic exercise (50)) and pharmacotherapies (e.g., statins (51), PCSK9 inhibitors (52), GLP-1 receptor agonists (53)), have been associated with modulation of ceramide or related metabolic pathways, but whether these effects translate into changes in CAC remains uncertain. Finally, mechanistic studies using disease models are urgently needed to decipher the molecular crosstalk between ceramides and CKD in relation to vascular calcification and to determine whether sphingolipid metabolism represents a potentially modifiable pathway.

This study has several limitations that merit consideration. First, the single-center design, despite rigorous multivariable adjustment, may limit external validity due to regional practice patterns and a relatively homogeneous patient population. Second, the cross-sectional nature precludes determination of the temporal or causal relationship between circulating ceramides and CAC. Although we identified associations between specific ceramides and calcification severity, longitudinal data are required to determine whether changes in ceramide levels precede changes in calcification burden. Third, while qualitative angiographic grading served as the primary assessment of calcification severity, we incorporated quantitative Agatston scoring from non-electrocardiogram-gated chest CT scans in a sensitivity analysis. This provided a more objective, reproducible measure of coronary calcium burden and partially mitigated the inherent subjectivity of angiographic grading. Moreover, imaging-based assessment cannot reliably distinguish atherosclerotic intimal calcification from CKD-associated medial calcification. Nevertheless, prospective use of coronary CTA is warranted. Finally, prevalent statin use (60.99%) and prior PCI (31.02%) could modulate ceramide levels and calcification interpretation. Residual confounding persists despite sensitivity analyses. Future research should prioritize multicenter longitudinal designs, quantitative imaging techniques, and treatment-naive populations to address these gaps.

## Conclusion

This study identifies specific circulating ceramide species associated with greater CAC severity and demonstrates that these associations are stronger in patients with CKD. The observed ceramide–kidney interaction, together with exploratory mediation findings, suggests a potentially relevant cardiorenal-metabolic link but does not establish temporal or causal relationships. Prospective longitudinal and interventional studies are needed to determine whether sphingolipid metabolism represents a modifiable pathway in CAC.

## Data Availability

The raw data supporting the conclusions of this article will be made available by the authors, without undue reservation.

## References

[1] Global Burden of Disease Study 2019 . Global burden of 369 diseases and injuries in 204 countries and territories, 1990-2019: a systematic analysis for the Global Burden of Disease Study 2019. Lancet (London England). (2020) 396:1204–22. doi: 10.1016/S0140-6736(20)30925-9 PMC7567026 33069326

[2] Durham AL Speer MY Scatena M Giachelli CM Shanahan CM . Role of smooth muscle cells in vascular calcification: implications in atherosclerosis and arterial stiffness. Cardiovasc Res. (2018) 114:590–600. doi: 10.1093/cvr/cvy010 29514202 PMC5852633

[3] Barbato E Gallinoro E Abdel-Wahab M Andreini D Carrié D Di Mario C . Management strategies for heavily calcified coronary stenoses: an EAPCI clinical consensus statement in collaboration with the EURO4C-PCR group. Eur Heart J. (2023) 44:4340–56. doi: 10.1093/eurheartj/ehad342 37208199

[4] Généreux P Madhavan MV Mintz GS Maehara A Palmerini T Lasalle L . Ischemic outcomes after coronary intervention of calcified vessels in acute coronary syndromes. Pooled analysis from the HORIZONS-AMI (Harmonizing Outcomes With Revascularization and Stents in Acute Myocardial Infarction) and ACUITY (Acute Catheterization and Urgent Intervention Triage Strategy) TRIALS. J Am Coll Cardiol. (2014) 63:1845–54. doi: 10.1016/j.jacc.2014.01.034 24561145

[5] Généreux P Redfors B Witzenbichler B Arsenault MP Weisz G Stuckey TD . Two-year outcomes after percutaneous coronary intervention of calcified lesions with drug-eluting stents. Int J Cardiol. (2017) 231:61–7. doi: 10.1016/j.ijcard.2016.12.150 28040289

[6] Raggi P Shaw LJ Berman DS Callister TQ . Prognostic value of coronary artery calcium screening in subjects with and without diabetes. J Am Coll Cardiol. (2004) 43:1663–9. doi: 10.1016/j.jacc.2003.09.068 15120828

[7] Kinde B Smith LD Vagefi MR . Exuberant scleral calcification from secondary hyperparathyroidism. Ophthalmology. (2022) 129:413. doi: 10.1016/j.ophtha.2021.10.026 35314032

[8] Jankowski J Floege J Fliser D Böhm M Marx N . Cardiovascular disease in chronic kidney disease: pathophysiological insights and therapeutic options. Circulation. (2021) 143:1157–72. doi: 10.1161/circulationaha.120.050686 33720773 PMC7969169

[9] Palit S Kendrick J . Vascular calcification in chronic kidney disease: role of disordered mineral metabolism. Curr Pharm Des. (2014) 20:5829–33. doi: 10.2174/1381612820666140212194926 24533939 PMC4130905

[10] Ketteler M Evenepoel P Holden RM Isakova T Jørgensen HS Komaba H . Chronic kidney disease-mineral and bone disorder: conclusions from a Kidney Disease: Improving Global Outcomes (KDIGO) Controversies Conference. Kidney Int. (2025) 107:405–23. doi: 10.1016/j.kint.2024.11.013 39864017

[11] Xu C Smith ER Tiong MK Ruderman I Toussaint ND . Interventions to attenuate vascular calcification progression in chronic kidney disease: a systematic review of clinical trials. J Am Soc Nephrol JASN. (2022) 33:1011–32. doi: 10.1681/asn.2021101327 35232774 PMC9063901

[12] Chertow GM Burke SK Raggi P . Sevelamer attenuates the progression of coronary and aortic calcification in hemodialysis patients. Kidney Int. (2002) 62:245–52. doi: 10.1046/j.1523-1755.2002.00434.x 12081584

[13] Henein MY Owen A . Statins moderate coronary stenoses but not coronary calcification: results from meta-analyses. Int J Cardiol. (2011) 153:31–5. doi: 10.1016/j.ijcard.2010.08.031 20843566

[14] Mitrofanova A Merscher S Fornoni A . Kidney lipid dysmetabolism and lipid droplet accumulation in chronic kidney disease. Nat Rev Nephrol. (2023) 19:629–45. doi: 10.1038/s41581-023-00741-w 37500941 PMC12926870

[15] Sun ZJ Chang DY Chen M Zhao MH . Deficiency of CFB attenuates renal tubulointerstitial damage by inhibiting ceramide synthesis in diabetic kidney disease. JCI Insight. (2022) 7:e156748. doi: 10.1172/jci.insight.156748 36546481 PMC9869976

[16] Baek J He C Afshinnia F Michailidis G Pennathur S . Lipidomic approaches to dissect dysregulated lipid metabolism in kidney disease. Nat Rev Nephrol. (2022) 18:38–55. doi: 10.1038/s41581-021-00488-2 PMC9146017 34616096

[17] Choi RH Tatum SM Symons JD Summers SA Holland WL . Ceramides and other sphingolipids as drivers of cardiovascular disease. Nat Rev Cardiol. (2021) 18:701–11. doi: 10.1038/s41569-021-00536-1 33772258 PMC8978615

[18] Zhang S Lin H Wang J Rui J Wang T Cai Z . Sensing ceramides by CYSLTR2 and P2RY6 to aggravate atherosclerosis. Nature. (2025) 641:476–85. doi: 10.1038/s41586-025-08792-8 40049228

[19] Liang L Li D Zeng R Zhang H Lv L Wei W . Long- and very long-chain ceramides are predictors of acute kidney injury in patients with acute coronary syndrome: the PEACP study. Cardiovasc Diabetol. (2023) 22:92. doi: 10.1186/s12933-023-01831-6 37081501 PMC10120114

[20] Pan W Li L Sun M Wang C Fang S Yu B . Plasma ceramides are associated with coronary atherosclerotic burden in patients with ST-segment elevation myocardial infarction. Int J Cardiol. (2020) 320:155–60. doi: 10.1016/j.ijcard.2020.08.010 32800902

[21] Laaksonen R Ekroos K Sysi-Aho M Hilvo M Vihervaara T Kauhanen D . Plasma ceramides predict cardiovascular death in patients with stable coronary artery disease and acute coronary syndromes beyond LDL-cholesterol. Eur Heart J. (2016) 37:1967–76. doi: 10.1093/eurheartj/ehw148 27125947 PMC4929378

[22] Hilvo M Meikle PJ Pedersen ER Tell GS Dhar I Brenner H . Development and validation of a ceramide- and phospholipid-based cardiovascular risk estimation score for coronary artery disease patients. Eur Heart J. (2020) 41:371–80. doi: 10.1093/eurheartj/ehz387 31209498

[23] Luong TTD Tuffaha R Schuchardt M Moser B Schelski N Boehme B . Acid sphingomyelinase promotes SGK1-dependent vascular calcification. Clin Sci (London Engl 1979). (2021) 135:515–34. doi: 10.1042/cs20201122 33479769 PMC7859357

[24] Song Y Hou M Li Z Luo C Ou JS Yu H . TLR4/NF-κB/ceramide signaling contributes to Ox-LDL-induced calcification of human vascular smooth muscle cells. Eur J Pharmacol. (2017) 794:45–51. doi: 10.1016/j.ejphar.2016.11.029 27876618

[25] Liao L Zhou Q Song Y Wu W Yu H Wang S . Ceramide mediates Ox-LDL-induced human vascular smooth muscle cell calcification via p38 mitogen-activated protein kinase signaling. PloS One. (2013) 8:e82379. doi: 10.1371/journal.pone.0082379 24358176 PMC3865066

[26] Chen Q Li Y He H Xie Y Fu D Gao R . Joint association of plasma ceramide and renal function with coronary artery lesion severity: the CRUISE-MET study. J Clin Lipidology. (2025) 19:1586–97. doi: 10.1016/j.jacl.2025.09.002 41038718

[27] Chen Q Li X Xie Y Li Y Sheng Z Che W . Chronic kidney disease, circulating ceramides, and coronary microvascular dysfunction: insights from the CRUISE-MET study. Cardiorenal Med. (2026) 16:321–33. doi: 10.1159/000552268 42060543 PMC13327663

[28] Cardiovascular-Renal Adverse oUtcome rIsk aSsessment systEm for Coronary Heart Disease Complicated With Chronic Kidney Disease Based on Targeted Lipid METabolomics. Available online at: https://clinicaltrials.gov/study/NCT06383208 (Accessed August 20, 2026).

[29] KDIGO . 2024 clinical practice guideline for the evaluation and management of chronic kidney disease. Kidney Int. (2024) 105:S117–314. doi: 10.1016/j.kint.2023.10.018 38490803

[30] Inker LA Eneanya ND Coresh J Tighiouart H Wang D Sang Y . New creatinine- and cystatin C-based equations to estimate GFR without race. N Engl J Med. (2021) 385:1737–49. doi: 10.1056/nejmoa2102953 34554658 PMC8822996

[31] Vrints C Andreotti F Koskinas KC Rossello X Adamo M Ainslie J . 2024 ESC Guidelines for the management of chronic coronary syndromes. Eur Heart J. (2024) 45:3415–537. doi: 10.1093/eurheartj/ehae177 39210710

[32] Torta F Hoffmann N Burla B Alecu I Arita M Bamba T . Concordant inter-laboratory derived concentrations of ceramides in human plasma reference materials via authentic standards. Nat Commun. (2024) 15:8562. doi: 10.1038/s41467-024-52087-x 39362843 PMC11449902

[33] Jiang H Hsu FF Farmer MS Peterson LR Schaffer JE Ory DS . Development and validation of LC-MS/MS method for determination of very long acyl chain (C22:0 and C24:0) ceramides in human plasma. Anal Bioanal Chem. (2013) 405:7357–65. doi: 10.1007/s00216-013-7166-9 23857140 PMC3766747

[34] Luque-Córdoba D Calderón-Santiago M Rangel-Zúñiga OA Camargo A López-Miranda J Priego-Capote F . Comprehensive profiling of ceramides in human serum by liquid chromatography coupled to tandem mass spectrometry combining data independent/dependent acquisition modes. Anal Chim Acta. (2024) 1287:342115. doi: 10.1016/j.aca.2023.342115 38182388

[35] Xu Y Li H Han Y Wang T Wang Y Gong J . A simple and rapid method for extraction and measurement of circulating sphingolipids using LC-MS/MS: a targeted lipidomic analysis. Anal Bioanal Chem. (2022) 414:2041–54. doi: 10.1007/s00216-021-03853-z 35066602

[36] Uai Discover Nongatedcacs. Available online at: https://www.uii-ai.com/product/12.html. (Accessed August 20, 2026)

[37] Li M Ling R Yu L Yang W Chen Z Wu D . Deep learning segmentation and reconstruction for CT of chronic total coronary occlusion. Radiology. (2023) 306:e221393. doi: 10.1148/radiol.221393 36283114

[38] Peng AW Dudum R Jain SS Maron DJ Patel BN Khandwala N . Association of coronary artery calcium detected by routine ungated CT imaging with cardiovascular outcomes. J Am Coll Cardiol. (2023) 82:1192–202. doi: 10.1016/j.jacc.2023.06.040 37704309 PMC11009374

[39] Leopold JA . Vascular calcification: an age-old problem of old age. Circulation. (2013) 127:2380–2. doi: 10.1161/CIRCULATIONAHA.113.003341 PMC3761061 23690467

[40] Nicoll R Zhao Y Ibrahimi P Olivecrona G Henein M . Diabetes and hypertension consistently predict the presence and extent of coronary artery calcification in symptomatic patients: a systematic review and meta-analysis. Int J Mol Sci. (2016) 17:1481. doi: 10.3390/ijms17091481 27608015 PMC5037759

[41] Henein M Granåsen G Wiklund U Schmermund A Guerci A Erbel R . High dose and long-term statin therapy accelerate coronary artery calcification. Int J Cardiol. (2015) 184:581–6. doi: 10.1016/j.ijcard.2015.02.072 25769003

[42] Ngamdu KS Ghosalkar DS Chung HE Christensen JL Lee C Butler CA . Long-term statin therapy is associated with severe coronary artery calcification. PloS One. (2023) 18:e0289111. doi: 10.1371/journal.pone.0289111 37498869 PMC10374064

[43] Hutcheson JD Goettsch C Bertazzo S Maldonado N Ruiz JL Goh W . Genesis and growth of extracellular-vesicle-derived microcalcification in atherosclerotic plaques. Nat Mater. (2016) 15:335–43. doi: 10.1038/nmat4519 26752654 PMC4767675

[44] Ceneri N Zhao L Young BD Healy A Coskun S Vasavada H . Rac2 modulates atherosclerotic calcification by regulating macrophage interleukin-1β production. Arteriosclerosis Thrombosis Vasc Biol. (2017) 37:328–40. doi: 10.1161/atvbaha.116.308507 27834690 PMC5269510

[45] Aikawa E Nahrendorf M Figueiredo JL Swirski FK Shtatland T Kohler RH . Osteogenesis associates with inflammation in early-stage atherosclerosis evaluated by molecular imaging *in vivo*. Circulation. (2007) 116:2841–50. doi: 10.1161/circulationaha.107.732867 18040026

[46] Chaurasia B Summers SA . Ceramides in metabolism: key lipotoxic players. Annu Rev Physiol. (2021) 83:303–30. doi: 10.1146/annurev-physiol-031620-093815 33158378 PMC7905841

[47] Bismuth J Lin P Yao Q Chen C . Ceramide: a common pathway for atherosclerosis? Atherosclerosis. (2008) 196:497–504. doi: 10.1016/j.atherosclerosis.2007.09.018 17963772 PMC2924671

[48] Huwiler A Pfeilschifter J . Sphingolipid signaling in renal fibrosis. Matrix Biol J Int Soc For Matrix Biol. (2018) 68-69:230–47. doi: 10.1016/j.matbio.2018.01.006 29343457

[49] Wang DD Toledo E Hruby A Rosner BA Willett WC Sun Q . Plasma ceramides, Mediterranean diet, and incident cardiovascular disease in the PREDIMED trial (Prevención con Dieta Mediterránea). Circulation. (2017) 135:2028–40. doi: 10.1161/circulationaha.116.024261 28280233 PMC5496817

[50] Parsons C Agasthi P Mookadam F Arsanjani R . Reversal of coronary atherosclerosis: role of life style and medical management. Trends Cardiovasc Med. (2018) 28:524–31. doi: 10.1016/j.tcm.2018.05.002 29807666

[51] Ng TW Ooi EM Watts GF Chan DC Weir JM Meikle PJ . Dose-dependent effects of rosuvastatin on the plasma sphingolipidome and phospholipidome in the metabolic syndrome. J Clin Endocrinol Metab. (2014) 99:E2335–40. doi: 10.1210/jc.2014-1665 25140396

[52] Hilvo M Simolin H Metso J Ruuth M Öörni K Jauhiainen M . PCSK9 inhibition alters the lipidome of plasma and lipoprotein fractions. Atherosclerosis. (2018) 269:159–65. doi: 10.1016/j.atherosclerosis.2018.01.004 29366988

[53] Wretlind A Curovic VR de Zawadzki A Suvitaival T Xu J Zobel EH . Ceramides are decreased after liraglutide treatment in people with type 2 diabetes: a post hoc analysis of two randomized clinical trials. Lipids Health Dis. (2023) 22:160. doi: 10.1186/s12944-023-01922-z 37752566 PMC10521385

