# Supplementary material for: Sphingolipid–renal interplay in coronary artery calcification: ceramide profiling and imaging validation from the CRUISE-MET study
Source: Front Endocrinol (Lausanne). 2026 Oct 14;17:1974775. doi: 10.3389/fendo.2026.1974775 (PMC13713281; doi:10.3389/fendo.2026.1974775)
Supplement: Supplementary file 1 [file SupplementaryFile1.docx]

**Supplementary Table S1. Baseline characteristics according to four-category angiographic coronary artery calcification severity**

| Variables | None  (n = 265) | Mild  (n = 222) | Moderate  (n = 181) | Severe  (n = 183) | *p* |
| --- | --- | --- | --- | --- | --- |
| Age, years | 60 ± 11 | 64 ± 10 | 65 ± 9 | 67 ± 9 | <.001 |
| Female, n (%) | 64 (24.15) | 58 (26.13) | 46 (25.41) | 61 (33.33) | 0.162 |
| BMI, kg/m² | 26.69 ± 4.62 | 26.42 ± 3.99 | 25.82 ± 3.65 | 25.40 ± 2.92 | 0.003 |
| Hypertension, n (%) | 201 (75.85) | 184 (82.88) | 149 (82.32) | 151 (82.51) | 0.151 |
| Diabetes mellitus, n (%) | 147 (55.47) | 132 (59.46) | 114 (62.98) | 139 (75.96) | <.001 |
| Smoking, n (%) | 123 (46.42) | 93 (41.89) | 85 (47.49) | 70 (38.89) | 0.282 |
| Previous PCI, n (%) | 92 (34.72) | 63 (28.38) | 56 (30.94) | 53 (28.96) | 0.427 |
| SBP, mmHg | 134 ± 21 | 136 ± 23 | 136 ± 21 | 135 ± 21 | 0.782 |
| HR, bpm | 77 ± 14 | 79 ± 15 | 77 ± 14 | 78 ± 14 | 0.476 |
| Hb, g/L | 139 ± 22 | 136 ± 22 | 133 ± 21 | 124 ± 24 | <.001 |
| FBG, mmol/L | 8.83 ± 4.50 | 9.22 ± 6.15 | 8.56 ± 4.02 | 9.44 ± 4.33 | 0.289 |
| HbA1c, % | 6.88 ± 1.42 | 7.04 ± 1.58 | 7.03 ± 1.44 | 7.20 ± 1.54 | 0.162 |
| TG, mmol/L | 1.59 (1.15, 2.21) | 1.66 (1.13, 2.35) | 1.58 (1.11, 2.28) | 1.48 (1.04, 2.28) | 0.670 |
| TC, mmol/L | 3.93 (3.19, 4.92) | 3.93 (3.15, 5.03) | 4.15 (3.12, 5.13) | 3.74 (3.00, 4.56) | 0.079 |
| LDL-C, mmol/L | 2.27 (1.80, 3.07) | 2.37 (1.84, 3.15) | 2.47 (1.87, 3.22) | 2.15 (1.73, 2.83) | 0.077 |
| HDL-C, mmol/L | 1.06 (0.90, 1.22) | 1.06 (0.91, 1.25) | 1.11 (0.94, 1.31) | 1.09 (0.89, 1.27) | 0.233 |
| Ca²⁺, mmol/L | 2.29 ± 0.13 | 2.30 ± 0.14 | 2.31 ± 0.15 | 2.29 ± 0.15 | 0.611 |
| P, mmol/L | 1.11 ± 0.23 | 1.15 ± 0.25 | 1.20 ± 0.33 | 1.22 ± 0.33 | <.001 |
| UACR, mg/g | 6.14 (0.17, 25.97) | 9.03 (0.59, 47.52) | 10.04 (0.31, 66.96) | 14.00 (0.42, 82.43) | 0.016 |
| eGFR, mL/min/1.73 m² | 91.37 (76.70, 104.45) | 86.35 (57.84, 99.12) | 83.45 (47.62, 95.78) | 68.58 (41.30, 89.67) | <.001 |
| CKD, n (%) | 81 (30.57) | 97 (43.69) | 85 (46.96) | 103 (56.28) | <.001 |
| bSS | 12 (7, 18) | 17 (10, 27) | 24 (14, 32) | 29 (19, 43) | <.001 |
| Complex CAD | 38 (14.34) | 38 (14.34) | 38 (14.34) | 38 (14.34) | <.001 |
| LVEF, % | 63 ± 9 | 60 ± 12 | 62 ± 10 | 61 ± 10 | 0.026 |
| Cer (18:1/16:0), ng/mL | 338.99 (274.30, 430.17) | 355.14 (275.31, 451.29) | 390.35 (279.99, 506.70) | 399.30 (314.41, 522.29) | <.001 |
| Cer (18:1/18:0), ng/mL | 81.56 (56.56, 106.43) | 79.85 (52.78, 108.10) | 87.10 (57.29, 126.09) | 79.53 (59.41, 125.50) | 0.120 |
| Cer (18:1/20:0), ng/mL | 51.80 (37.10, 73.10) | 53.46 (35.44, 71.51) | 56.69 (34.20, 86.28) | 53.74 (36.91, 78.23) | 0.490 |
| Cer (18:1/22:0), ng/mL | 5880.87 (4386.54, 8264.81) | 6108.61 (4334.34, 8073.92) | 6215.65 (4376.54, 9197.54) | 5694.73 (4357.35, 8106.98) | 0.706 |
| Cer (18:1/24:0), ng/mL | 1348.87 (974.47, 1748.46) | 1368.65 (948.53, 1851.00) | 1265.62 (979.45, 1813.54) | 1324.39 (958.34, 1815.58) | 0.964 |
| Cer (18:1/24:1), ng/mL | 649.34 (471.45, 862.52) | 664.27 (472.51, 825.44) | 741.20 (528.08, 987.56) | 673.88 (520.24, 892.77) | 0.011 |
| **Medication history** |  |  |  |  |  |
| Antiplatelet drugs, n (%) | 175 (66.04) | 139 (62.61) | 118 (65.19) | 126 (68.85) | 0.621 |
| Statins, n (%) | 168 (63.40) | 140 (63.06) | 107 (59.12) | 104 (56.83) | 0.450 |
| Ezetimibe, n (%) | 77 (29.06) | 64 (28.83) | 48 (26.52) | 53 (28.96) | 0.936 |
| PCSK-9i, n (%) | 40 (15.09) | 23 (10.36) | 29 (16.02) | 20 (10.93) | 0.214 |

Data are presented as mean ± standard deviation, median (interquartile range), or n (%), as appropriate. CAC severity was classified angiographically as absent, mild, moderate, or severe, as defined in the Methods. **Abbreviations:** CAC, coronary artery calcification; BMI, body mass index; SBP, systolic blood pressure; HR, heart rate; FBG, fasting blood glucose; FBG, fasting blood glucose; HbA1c, glycated hemoglobin; LVEF, left ventricular ejection fraction; Hb, hemoglobin; UACR, urine albumin-to-creatinine ratio; eGFR, estimated glomerular filtration rate; bSS, baseline SYNTAX score; CKD, chronic kidney disease; PCI, percutaneous coronary intervention; TG, triglycerides; TC, total cholesterol; LDL-C, low-density lipoprotein cholesterol; HDL-C, high-density lipoprotein cholesterol; Cer, ceramide.

**Table S2. Univariable Logistic Regression Analysis**

| Variables | β | S.E | Z | *p* | OR (95%CI) |
| --- | --- | --- | --- | --- | --- |
|  |  |  |  |  |  |
| Age | 0.04 | 0.01 | 5.41 | **<.001** | 1.04 (1.03 ~ 1.05) |
| Sex | 0.22 | 0.16 | 1.41 | 0.158 | 1.25 (0.92 ~ 1.69) |
| BMI | -0.07 | 0.02 | -3.44 | **<.001** | 0.94 (0.90 ~ 0.97) |
| Previous PCI | -0.09 | 0.15 | -0.59 | 0.557 | 0.92 (0.68 ~ 1.23) |
| Hypertension | 0.22 | 0.18 | 1.22 | 0.221 | 1.24 (0.88 ~ 1.76) |
| Diabetes mellitus | 0.53 | 0.15 | 3.63 | **<.001** | 1.70 (1.28 ~ 2.26) |
| Smoking | -0.05 | 0.14 | -0.34 | 0.733 | 0.95 (0.72 ~ 1.25) |
| Previous antiplatelet agents | 0.11 | 0.15 | 0.78 | 0.437 | 1.12 (0.84 ~ 1.49) |
| Previous statin use | -0.22 | 0.14 | -1.56 | 0.119 | 0.80 (0.61 ~ 1.06) |
| CKD | 0.62 | 0.14 | 4.38 | **<.001** | 1.85 (1.41 ~ 2.44) |
| TG | 0.01 | 0.04 | 0.30 | 0.762 | 1.01 (0.93 ~ 1.11) |
| LDLC | -0.02 | 0.07 | -0.32 | 0.749 | 0.98 (0.84 ~ 1.13) |
| HDLC | 0.53 | 0.25 | 2.16 | **0.031** | 1.71 (1.05 ~ 2.78) |
| Ca | -0.06 | 0.50 | -0.12 | 0.904 | 0.94 (0.36 ~ 2.49) |
| P | 0.96 | 0.26 | 3.69 | **<.001** | 2.60 (1.57 ~ 4.33) |
| OR: Odds Ratio, CI: Confidence Interval | | | | | |

**Table S3. Logistic Regression Analysis of Plasma Ceramide Concentrations and Coronary Calcification**

|  | Model I | |  | Model II | |  | Model III | |
| --- | --- | --- | --- | --- | --- | --- | --- | --- |
|  | OR (95% CI) | *p* |  | OR (95% CI) | *p* |  | OR (95% CI) | *p* |
| Cer (18:1/20:0) (per SD) | 1.18 (1.03 ~ 1.36) | 0.019 |  | 1.31 (1.13 ~ 1.52) | <.001 |  | 1.32 (1.11 ~ 1.56) | 0.001 |
| Cer (18:1/20:0) (tertiles) |  |  |  |  |  |  |  |  |
| T1 | 1.00 (Reference) |  |  | 1.00 (Reference) |  |  | 1.00 (Reference) |  |
| T2 | 0.85 (0.61 ~ 1.19) | 0.337 |  | 0.92 (0.65 ~ 1.31) | 0.658 |  | 0.88 (0.62 ~ 1.26) | 0.495 |
| T3 | 1.19 (0.85 ~ 1.66) | 0.309 |  | 1.47 (1.03 ~ 2.09) | 0.034 |  | 1.38 (0.94 ~ 2.02) | 0.098 |
| *p* value for trend | 0.230 |  |  | 0.022 |  |  | 0.073 |  |
| Cer (18:1/22:0) (per SD) | 1.05 (0.92 ~ 1.20) | 0.493 |  | 1.15 (0.99 ~ 1.33) | 0.065 |  | 1.16 (0.97 ~ 1.39) | 0.097 |
| Cer (18:1/22:0) (tertiles) |  |  |  |  |  |  |  |  |
| T1 | 1.00 (Reference) |  |  | 1.00 (Reference) |  |  | 1.00 (Reference) |  |
| T2 | 0.85 (0.61 ~ 1.18) | 0.336 |  | 0.85 (0.60 ~ 1.21) | 0.373 |  | 0.82 (0.57 ~ 1.17) | 0.268 |
| T3 | 1.04 (0.75 ~ 1.46) | 0.799 |  | 1.18 (0.83 ~ 1.68) | 0.365 |  | 1.19 (0.80 ~ 1.76) | 0.396 |
| *p* value for trend | 0.664 |  |  | 0.269 |  |  | 0.312 |  |
| Cer (18:1/24:0) (per SD) | 1.01 (0.88 ~ 1.16) | 0.894 |  | 1.11 (0.95 ~ 1.28) | 0.180 |  | 1.12 (0.93 ~ 1.35) | 0.221 |
| Cer (18:1/24:0) (tertiles) |  |  |  |  |  |  |  |  |
| T1 | 1.00 (Reference) |  |  | 1.00 (Reference) |  |  | 1.00 (Reference) |  |
| T2 | 0.97 (0.69 ~ 1.34) | 0.833 |  | 0.99 (0.70 ~ 1.40) | 0.968 |  | 0.95 (0.66 ~ 1.36) | 0.765 |
| T3 | 0.88 (0.63 ~ 1.23) | 0.443 |  | 0.98 (0.69 ~ 1.40) | 0.911 |  | 0.97 (0.64 ~ 1.46) | 0.882 |
| *p* value for trend | 0.436 |  |  | 0.911 |  |  | 0.885 |  |

Model1: Crude

Model2: Adjusted for sex, age, BMI, previous PCI, hypertension, diabetes mellitus, smoking

Model3: Adjusted for sex, age, BMI, previous PCI, hypertension, diabetes mellitus, smoking, CKD, TG, LDL-C, HDL-C, Ca2+, P, antiplatelet agents use and statins use.

Abbreviations: BMI, body mass index; TG, triglyceride; HDL-C, high density lipoprotein cholesterol; LDL-C, low density lipoprotein cholesterol; CKD, chronic kidney disease; Ca^2+^, serum calcium; P, serum phosphorus; CI, confidence interval; OR, odds ratio; SD, standard deviation.

**Table S4. Baseline characteristics Stratified by CACS**

| Variables | None  (n = 43) | Mild  (n = 58) | Moderate  (n = 66) | Severe  (n = 113) | P |
| --- | --- | --- | --- | --- | --- |
|  |  |  |  |  |  |
| Age, years | 60.88 ± 12.76 | 62.86 ± 10.51 | 64.50 ± 9.82 | 66.66 ± 9.02 | 0.008 |
| Female, n (%) | 12 (27.91) | 14 (24.14) | 28 (42.42) | 36 (31.86) | 0.154 |
| BMI, kg/m2 | 26.88 ± 5.36 | 26.76 ± 3.68 | 26.66 ± 4.50 | 25.28 ± 2.93 | 0.025 |
| Hypertension, n (%) | 34 (79.07) | 50 (86.21) | 54 (81.82) | 92 (81.42) | 0.807 |
| Diabetes mellitus, n (%) | 20 (46.51) | 32 (55.17) | 37 (56.06) | 82 (72.57) | 0.009 |
| Smoking, n (%) | 15 (34.88) | 29 (50.88) | 22 (33.33) | 44 (39.64) | 0.212 |
| Hb, g/L | 143.62 ± 18.30 | 133.80 ± 20.05 | 128.77 ± 23.02 | 128.81 ± 23.12 | 0.001 |
| FBG, mmol/L | 8.92 ± 4.45 | 8.00 ± 3.45 | 8.53 ± 4.00 | 8.79 ± 3.98 | 0.592 |
| HbA1c, % | 6.77 ± 1.27 | 6.81 ± 1.43 | 6.89 ± 1.13 | 7.14 ± 1.61 | 0.352 |
| TG, mmol/L | 1.42 (1.07,2.23) | 1.42 (1.09,1.84) | 1.89 (1.19,2.47) | 1.61 (1.07,2.37) | 0.270 |
| TC, mmol/L | 4.18 (3.48,5.14) | 4.50 (3.31,5.38) | 4.48 (3.57,5.39) | 4.10 (3.35,4.81) | 0.293 |
| LDL-C, mmol/L | 2.56 (1.95,3.34) | 2.77 (2.09,3.38) | 2.63 (1.95,3.43) | 2.46 (1.95,3.09) | 0.434 |
| HDL-C, mmol/L | 1.09 (0.98,1.21) | 1.13 (0.90,1.29) | 1.15 (0.99,1.39) | 1.10 (0.94,1.33) | 0.490 |
| Ca2+, mmol/L | 2.32 ± 0.10 | 2.26 ± 0.12 | 2.30 ± 0.13 | 2.29 ± 0.18 | 0.186 |
| P, mmol/L | 1.10 ± 0.18 | 1.09 ± 0.23 | 1.20 ± 0.28 | 1.17 ± 0.30 | 0.072 |
| UACR, mg/g | 7.78 (0.26,26.83) | 7.06 (0.22,79.09) | 5.54 (0.21,21.46) | 10.32 (0.39,82.36) | 0.420 |
| eGFR, mL/min/1.73m2 | 90.89 (62.83,104.34) | 91.38 (64.64,97.89) | 83.20 (56.96,96.17) | 79.06 (46.11,93.51) | 0.009 |
| CKD, n (%) | 13 (30.23) | 21 (36.21) | 25 (37.88) | 56 (49.56) | 0.098 |
| bSS | 12.00 (6.00,17.00) | 15.50 (9.00,22.88) | 17.50 (12.12,25.00) | 24.00 (12.00,35.50) | <.001 |
| Cer (18:1/16:0), ng/mL | 338.99 (277.12,406.13) | 358.50 (285.10,457.73) | 377.26 (310.25,495.10) | 417.37 (320.84,528.34) | 0.008 |
| Cer (18:1/18:0), ng/mL | 78.31 (50.13,101.47) | 77.46 (55.51,103.88) | 87.43 (63.18,115.16) | 88.17 (63.94,118.97) | 0.051 |
| Cer (18:1/20:0), ng/mL | 49.94 (32.05,73.04) | 56.94 (35.02,70.66) | 56.53 (43.04,79.99) | 59.50 (38.18,80.73) | 0.244 |
| Cer (18:1/22:0), ng/mL | 6758.54 (4516.85,8501.61) | 6137.07 (4654.58,7696.33) | 7001.01 (4847.48,10101.40) | 6088.17 (4467.81,8333.82) | 0.420 |
| Cer (18:1/24:0), ng/mL | 1434.66 (1136.27,1804.69) | 1296.38 (992.64,1718.60) | 1511.67 (1077.79,1961.72) | 1382.57 (996.34,1862.19) | 0.627 |
| Cer (18:1/24:1), ng/mL | 621.08 (415.56,804.67) | 675.71 (472.98,990.44) | 702.15 (505.48,831.17) | 740.36 (571.47,941.10) | 0.095 |
| Antiplatelet drugs, n (%) | 26 (60.47) | 30 (51.72) | 29 (43.94) | 76 (67.26) | 0.016 |
| Statins, n (%) | 23 (53.49) | 36 (62.07) | 39 (59.09) | 69 (61.06) | 0.820 |
| Ezetimibe, n (%) | 8 (18.60) | 21 (36.21) | 15 (22.73) | 31 (27.43) | 0.198 |
| PCSK-9i, n (%) | 4 (9.30) | 6 (10.34) | 10 (15.15) | 17 (15.04) | 0.674 |
| Antidiabetic agents, n (%) | 18 (41.86) | 27 (47.37) | 32 (48.48) | 69 (62.16) | 0.068 |

**Abbreviations:** BMI, body mass index; PCI, percutaneous coronary intervention; FBG, fasting blood glucose; TG, triglyceride; TC, total cholesterol; HDL-C, high density lipoprotein cholesterol; LDL-C, low density lipoprotein cholesterol; UACR, urine albumin-to-creatinine ratio; eGFR, estimating glomerular filtration rate; CKD, chronic kidney disease; Ca^2+^, serum calcium; P, serum phosphorus; LVEF, left ventricular ejection fraction; Cer: Ceramide; bSS, baseline SYNTAX score; PCSK-9i, proprotein convertase subtilisin/kexin type 9 inhibitors; CACS, coronary artery calcium score.

**Table S5. Linear Regression Analysis of Plasma Ceramide Concentrations and CACS** **in Participants Underwent non-electrocardiogram-gated Chest CT scans**

|  | Model I | |  | Model II | |  | Model III | |
| --- | --- | --- | --- | --- | --- | --- | --- | --- |
|  | β (95% CI) | P |  | β (95% CI) | P |  | β (95% CI) | P |
| Cer (18:1/16:0) (per SD) | 0.23 (0.11 ~ 0.35) | <.001 |  | 0.24 (0.13 ~ 0.36) | <.001 |  | 0.23 (0.10 ~ 0.36) | <.001 |
| Cer (18:1/16:0) (tertiles) |  |  |  |  |  |  |  |  |
| T1 | 0.00 (Reference) |  |  | 0.00 (Reference) |  |  | 0.00 (Reference) |  |
| T2 | 0.10 (-0.21 ~ 0.41) | 0.532 |  | 0.09 (-0.21 ~ 0.39) | 0.570 |  | 0.07 (-0.23 ~ 0.38) | 0.632 |
| T3 | 0.41 (0.10 ~ 0.72) | 0.011 |  | 0.47 (0.16 ~ 0.77) | 0.003 |  | 0.40 (0.07 ~ 0.74) | 0.019 |
| P value for trend | 0.009 |  |  | 0.002 |  |  | 0.017 |  |
| Cer (18:1/18:0) (per SD) | 0.14 (0.02 ~ 0.27) | 0.021 |  | 0.21 (0.09 ~ 0.33) | <.001 |  | 0.18 (0.05 ~ 0.31) | 0.007 |
| Cer (18:1/18:0) (tertiles) |  |  |  |  |  |  |  |  |
| T1 | 0.00 (Reference) |  |  | 0.00 (Reference) |  |  | 0.00 (Reference) |  |
| T2 | 0.38 (0.07 ~ 0.69) | 0.018 |  | 0.39 (0.09 ~ 0.69) | 0.011 |  | 0.38 (0.08 ~ 0.69) | 0.014 |
| T3 | 0.36 (0.05 ~ 0.67) | 0.023 |  | 0.46 (0.15 ~ 0.77) | 0.004 |  | 0.38 (0.05 ~ 0.70) | 0.024 |
| P value for trend | 0.034 |  |  | 0.005 |  |  | 0.034 |  |
| Cer (18:1/20:0) (per SD) | 0.14 (-0.00 ~ 0.27) | 0.056 |  | 0.19 (0.06 ~ 0.33) | 0.005 |  | 0.15 (-0.00 ~ 0.30) | 0.052 |
| Cer (18:1/20:0) (tertiles) |  |  |  |  |  |  |  |  |
| T1 | 0.00 (Reference) |  |  | 0.00 (Reference) |  |  | 0.00 (Reference) |  |
| T2 | 0.16 (-0.15 ~ 0.47) | 0.311 |  | 0.23 (-0.07 ~ 0.54) | 0.135 |  | 0.21 (-0.09 ~ 0.52) | 0.174 |
| T3 | 0.25 (-0.06 ~ 0.56) | 0.116 |  | 0.36 (0.06 ~ 0.67) | 0.021 |  | 0.24 (-0.09 ~ 0.57) | 0.152 |
| P value for trend | 0.122 |  |  | 0.023 |  |  | 0.169 |  |
| Cer (18:1/22:0) (per SD) | -0.03 (-0.17 ~ 0.12) | 0.724 |  | 0.04 (-0.10 ~ 0.18) | 0.579 |  | -0.00 (-0.16 ~ 0.16) | 0.980 |
| Cer (18:1/22:0) (tertiles) |  |  |  |  |  |  |  |  |
| T1 | 0.00 (Reference) |  |  | 0.00 (Reference) |  |  | 0.00 (Reference) |  |
| T2 | -0.06 (-0.37 ~ 0.25) | 0.709 |  | -0.00 (-0.31 ~ 0.30) | 0.979 |  | -0.01 (-0.32 ~ 0.30) | 0.956 |
| T3 | -0.05 (-0.36 ~ 0.27) | 0.771 |  | 0.06 (-0.25 ~ 0.37) | 0.709 |  | 0.01 (-0.32 ~ 0.34) | 0.954 |
| P value for trend | 0.801 |  |  | 0.686 |  |  | 0.945 |  |
| Cer (18:1/24:0) (per SD) | -0.02 (-0.15 ~ 0.11) | 0.755 |  | 0.04 (-0.09 ~ 0.17) | 0.531 |  | 0.02 (-0.13 ~ 0.18) | 0.771 |
| Cer (18:1/24:0) (tertiles) |  |  |  |  |  |  |  |  |
| T1 | 0.00 (Reference) |  |  | 0.00 (Reference) |  |  | 0.00 (Reference) |  |
| T2 | -0.36 (-0.67 ~ -0.05) | 0.023 |  | -0.31 (-0.61 ~ -0.01) | 0.045 |  | -0.31 (-0.62 ~ 0.00) | 0.052 |
| T3 | -0.22 (-0.53 ~ 0.09) | 0.164 |  | -0.10 (-0.40 ~ 0.21) | 0.541 |  | -0.15 (-0.49 ~ 0.20) | 0.409 |
| P value for trend | 0.227 |  |  | 0.671 |  |  | 0.488 |  |
| Cer (18:1/24:1) (per SD) | 0.14 (0.01 ~ 0.27) | 0.031 |  | 0.19 (0.07 ~ 0.32) | 0.002 |  | 0.17 (0.03 ~ 0.31) | 0.019 |
| Cer (18:1/24:1) (tertiles) |  |  |  |  |  |  |  |  |
| T1 | 0.00 (Reference) |  |  | 0.00 (Reference) |  |  | 0.00 (Reference) |  |
| T2 | 0.25 (-0.06 ~ 0.56) | 0.119 |  | 0.31 (0.01 ~ 0.61) | 0.047 |  | 0.27 (-0.04 ~ 0.58) | 0.092 |
| T3 | 0.31 (0.01 ~ 0.63) | 0.049 |  | 0.39 (0.09 ~ 0.70) | 0.011 |  | 0.30 (-0.04 ~ 0.64) | 0.086 |
| P value for trend | 0.052 |  |  | 0.013 |  |  | 0.097 |  |

CI: Confidence Interval; CACS, coronary artery calcium score.

Model1: Crude

Model2: Adjusted for sex, age, BMI, HTN, DM, smoking

Model3: Adjusted for sex, age, BMI, HTN, DM, smoking, CKD, TG, LDL-C, HDL-C, Ca^2+^, P, antiplatelet agents, and statins.

**Table S6. Logistic Regression Analysis of Plasma Ceramide Concentrations and Coronary Calcification in the complete renal biomarker cohort**

|  | Model I | |  | Model II | |  | Model III | |
| --- | --- | --- | --- | --- | --- | --- | --- | --- |
|  | OR (95% CI) | P |  | OR (95% CI) | P |  | OR (95% CI) | P |
| Cer (18:1/16:0) (per SD) | 1.46 (1.25 ~ 1.71) | <.001 |  | 1.64 (1.38 ~ 1.95) | <.001 |  | 1.68 (1.37 ~ 2.04) | <.001 |
| Cer (18:1/16:0) (tertiles) |  |  |  |  |  |  |  |  |
| T1 | 1.00 (Reference) |  |  | 1.00 (Reference) |  |  | 1.00 (Reference) |  |
| T2 | 1.15 (0.79 ~ 1.67) | 0.473 |  | 1.17 (0.79 ~ 1.73) | 0.442 |  | 1.19 (0.79 ~ 1.79) | 0.411 |
| T3 | 2.00 (1.39 ~ 2.90) | <.001 |  | 2.57 (1.72 ~ 3.84) | <.001 |  | 2.59 (1.65 ~ 4.06) | <.001 |
| P value for trend | <.001 |  |  | <.001 |  |  | <.001 |  |
| Cer (18:1/18:0) (per SD) | 1.29 (1.11 ~ 1.50) | <.001 |  | 1.51 (1.28 ~ 1.79) | <.001 |  | 1.50 (1.25 ~ 1.80) | <.001 |
| Cer (18:1/18:0) (tertiles) |  |  |  |  |  |  |  |  |
| T1 | 1.00 (Reference) |  |  | 1.00 (Reference) |  |  | 1.00 (Reference) |  |
| T2 | 1.16 (0.80 ~ 1.67) | 0.434 |  | 1.29 (0.88 ~ 1.89) | 0.199 |  | 1.24 (0.83 ~ 1.85) | 0.284 |
| T3 | 1.44 (1.00 ~ 2.08) | 0.051 |  | 1.83 (1.23 ~ 2.71) | 0.003 |  | 1.75 (1.14 ~ 2.67) | 0.010 |
| P value for trend | 0.049 |  |  | 0.003 |  |  | 0.010 |  |
| Cer (18:1/24:1) (per SD) | 1.29 (1.11 ~ 1.50) | <.001 |  | 1.46 (1.24 ~ 1.72) | <.001 |  | 1.43 (1.19 ~ 1.73) | <.001 |
| Cer (18:1/24:1) (tertiles) |  |  |  |  |  |  |  |  |
| T1 | 1.00 (Reference) |  |  | 1.00 (Reference) |  |  | 1.00 (Reference) |  |
| T2 | 1.22 (0.85 ~ 1.77) | 0.284 |  | 1.24 (0.84 ~ 1.83) | 0.275 |  | 1.21 (0.81 ~ 1.80) | 0.355 |
| T3 | 1.60 (1.11 ~ 2.32) | 0.012 |  | 1.94 (1.31 ~ 2.88) | <.001 |  | 1.81 (1.17 ~ 2.80) | 0.008 |
| P value for trend | 0.012 |  |  | <.001 |  |  | 0.007 |  |

Model1: Crude

Model2: Adjusted for sex, age, BMI, previous PCI, hypertension, diabetes mellitus, smoking

Model3: Adjusted for sex, age, BMI, previous PCI, hypertension, diabetes mellitus, smoking, CKD, TG, LDL-C, HDL-C, Ca2+, P, antiplatelet agents use and statins use.

Abbreviations: BMI, body mass index; TG, triglyceride; HDL-C, high density lipoprotein cholesterol; LDL-C, low density lipoprotein cholesterol; CKD, chronic kidney disease; Ca^2+^, serum calcium; P, serum phosphorus; CI, confidence interval; OR, odds ratio; SD, standard deviation.

**Table S7. Logistic Regression Analysis of Plasma Ceramide Concentrations and Coronary Calcification in patients with a history of statin therapy**

|  | Model I | |  | Model II | |  | Model III | |
| --- | --- | --- | --- | --- | --- | --- | --- | --- |
|  | OR (95% CI) | P |  | OR (95% CI) | P |  | OR (95% CI) | P |
| Cer (18:1/16:0) (per SD) | 1.55 (1.28 ~ 1.87) | <.001 |  | 1.64 (1.34 ~ 2.01) | <.001 |  | 1.86 (1.46 ~ 2.38) | <.001 |
| Cer (18:1/16:0) (tertiles) |  |  |  |  |  |  |  |  |
| T1 | 1.00 (Reference) |  |  | 1.00 (Reference) |  |  | 1.00 (Reference) |  |
| T2 | 1.13 (0.73 ~ 1.76) | 0.584 |  | 1.13 (0.72 ~ 1.79) | 0.592 |  | 1.23 (0.77 ~ 1.97) | 0.386 |
| T3 | 2.07 (1.34 ~ 3.19) | 0.001 |  | 2.37 (1.49 ~ 3.78) | <.001 |  | 2.72 (1.61 ~ 4.60) | <.001 |
| P value for trend | <.001 |  |  | <.001 |  |  | <.001 |  |
| Cer (18:1/18:0) (per SD) | 1.41 (1.16 ~ 1.70) | <.001 |  | 1.58 (1.28 ~ 1.95) | <.001 |  | 1.67 (1.33 ~ 2.10) | <.001 |
| Cer (18:1/18:0) (tertiles) |  |  |  |  |  |  |  |  |
| T1 | 1.00 (Reference) |  |  | 1.00 (Reference) |  |  | 1.00 (Reference) |  |
| T2 | 1.07 (0.69 ~ 1.66) | 0.754 |  | 1.09 (0.69 ~ 1.72) | 0.716 |  | 1.12 (0.70 ~ 1.78) | 0.639 |
| T3 | 1.74 (1.13 ~ 2.69) | 0.012 |  | 1.94 (1.22 ~ 3.09) | 0.005 |  | 1.96 (1.19 ~ 3.23) | 0.008 |
| P value for trend | 0.008 |  |  | 0.003 |  |  | 0.006 |  |
| Cer (18:1/24:1) (per SD) | 1.34 (1.12 ~ 1.62) | 0.002 |  | 1.45 (1.19 ~ 1.77) | <.001 |  | 1.58 (1.25 ~ 2.00) | <.001 |
| Cer (18:1/24:1) (tertiles) |  |  |  |  |  |  |  |  |
| T1 | 1.00 (Reference) |  |  | 1.00 (Reference) |  |  | 1.00 (Reference) |  |
| T2 | 1.12 (0.73 ~ 1.74) | 0.596 |  | 1.09 (0.69 ~ 1.72) | 0.705 |  | 1.11 (0.69 ~ 1.78) | 0.659 |
| T3 | 1.66 (1.08 ~ 2.56) | 0.022 |  | 1.78 (1.12 ~ 2.80) | 0.014 |  | 1.85 (1.11 ~ 3.08) | 0.018 |
| P value for trend | 0.019 |  |  | 0.012 |  |  | 0.016 |  |

Model1: Crude

Model2: Adjusted for sex, age, BMI, previous PCI, hypertension, diabetes mellitus, smoking

Model3: Adjusted for sex, age, BMI, previous PCI, hypertension, diabetes mellitus, smoking, CKD, TG, LDL-C, HDL-C, Ca2+, P, and antiplatelet agents use.

Abbreviations: BMI, body mass index; TG, triglyceride; HDL-C, high density lipoprotein cholesterol; LDL-C, low density lipoprotein cholesterol; CKD, chronic kidney disease; Ca^2+^, serum calcium; P, serum phosphorus; CI, confidence interval; OR, odds ratio; SD, standard deviation.

**Table S8. Logistic Regression Analysis of Plasma Ceramide Concentrations and Coronary Calcification in patients with Diabetes Mellitus**

|  | Model I | |  | Model II | |  | Model III | |
| --- | --- | --- | --- | --- | --- | --- | --- | --- |
|  | OR (95% CI) | P |  | OR (95% CI) | P |  | OR (95% CI) | P |
| Cer (18:1/16:0) (per SD) | 1.49 (1.25 ~ 1.78) | <.001 |  | 1.56 (1.30 ~ 1.87) | <.001 |  | 1.58 (1.29 ~ 1.93) | <.001 |
| Cer (18:1/16:0) (tertiles) |  |  |  |  |  |  |  |  |
| T1 | 1.00 (Reference) |  |  | 1.00 (Reference) |  |  | 1.00 (Reference) |  |
| T2 | 1.39 (0.91 ~ 2.12) | 0.126 |  | 1.37 (0.89 ~ 2.12) | 0.154 |  | 1.41 (0.89 ~ 2.21) | 0.142 |
| T3 | 2.14 (1.40 ~ 3.27) | <.001 |  | 2.35 (1.51 ~ 3.65) | <.001 |  | 2.47 (1.52 ~ 4.01) | <.001 |
| P value for trend | <.001 |  |  | <.001 |  |  | <.001 |  |
| Cer (18:1/18:0) (per SD) | 1.28 (1.07 ~ 1.54) | 0.008 |  | 1.38 (1.14 ~ 1.67) | <.001 |  | 1.37 (1.11 ~ 1.68) | 0.003 |
| Cer (18:1/18:0) (tertiles) |  |  |  |  |  |  |  |  |
| T1 | 1.00 (Reference) |  |  | 1.00 (Reference) |  |  | 1.00 (Reference) |  |
| T2 | 1.00 (0.66 ~ 1.53) | 0.983 |  | 1.08 (0.70 ~ 1.66) | 0.740 |  | 1.09 (0.70 ~ 1.71) | 0.695 |
| T3 | 1.38 (0.90 ~ 2.09) | 0.136 |  | 1.55 (1.01 ~ 2.40) | 0.048 |  | 1.54 (0.97 ~ 2.46) | 0.068 |
| P value for trend | 0.120 |  |  | 0.041 |  |  | 0.061 |  |
| Cer (18:1/24:1) (per SD) | 1.26 (1.06 ~ 1.50) | 0.010 |  | 1.33 (1.11 ~ 1.60) | 0.002 |  | 1.31 (1.07 ~ 1.61) | 0.010 |
| Cer (18:1/24:1) (tertiles) |  |  |  |  |  |  |  |  |
| T1 | 1.00 (Reference) |  |  | 1.00 (Reference) |  |  | 1.00 (Reference) |  |
| T2 | 1.10 (0.72 ~ 1.67) | 0.654 |  | 1.09 (0.71 ~ 1.68) | 0.681 |  | 1.07 (0.68 ~ 1.67) | 0.768 |
| T3 | 1.44 (0.95 ~ 2.19) | 0.088 |  | 1.53 (0.99 ~ 2.36) | 0.055 |  | 1.43 (0.89 ~ 2.30) | 0.145 |
| P value for trend | 0.085 |  |  | 0.052 |  |  | 0.139 |  |

Model1: Crude

Model2: Adjusted for sex, age, BMI, previous PCI, hypertension, smoking

Model3: Adjusted for sex, age, BMI, previous PCI, hypertension, smoking, CKD, TG, LDL-C, HDL-C, Ca2+, P, antiplatelet agents use and statins use.

Abbreviations: BMI, body mass index; TG, triglyceride; HDL-C, high density lipoprotein cholesterol; LDL-C, low density lipoprotein cholesterol; CKD, chronic kidney disease; Ca^2+^, serum calcium; P, serum phosphorus; CI, confidence interval; OR, odds ratio; SD, standard deviation.

**Table S9. Logistic Regression Analysis of Plasma Ceramide Concentrations and Coronary Calcification in patients with comorbid Hypertension**

|  | Model I | |  | Model II | |  | Model III | |
| --- | --- | --- | --- | --- | --- | --- | --- | --- |
|  | OR (95% CI) | P |  | OR (95% CI) | P |  | OR (95% CI) | P |
| Cer (18:1/16:0) (per SD) | 1.44 (1.23 ~ 1.69) | <.001 |  | 1.49 (1.26 ~ 1.76) | <.001 |  | 1.48 (1.23 ~ 1.79) | <.001 |
| Cer (18:1/16:0) (tertiles) |  |  |  |  |  |  |  |  |
| T1 | 1.00 (Reference) |  |  | 1.00 (Reference) |  |  | 1.00 (Reference) |  |
| T2 | 1.12 (0.77 ~ 1.64) | 0.543 |  | 1.14 (0.77 ~ 1.69) | 0.526 |  | 1.10 (0.73 ~ 1.66) | 0.651 |
| T3 | 2.13 (1.46 ~ 3.11) | <.001 |  | 2.37 (1.60 ~ 3.53) | <.001 |  | 2.34 (1.51 ~ 3.62) | <.001 |
| P value for trend | <.001 |  |  | <.001 |  |  | <.001 |  |
| Cer (18:1/18:0) (per SD) | 1.24 (1.07 ~ 1.45) | 0.005 |  | 1.35 (1.14 ~ 1.59) | <.001 |  | 1.38 (1.16 ~ 1.65) | <.001 |
| Cer (18:1/18:0) (tertiles) |  |  |  |  |  |  |  |  |
| T1 | 1.00 (Reference) |  |  | 1.00 (Reference) |  |  | 1.00 (Reference) |  |
| T2 | 1.18 (0.81 ~ 1.71) | 0.388 |  | 1.23 (0.83 ~ 1.80) | 0.302 |  | 1.24 (0.83 ~ 1.86) | 0.286 |
| T3 | 1.46 (1.01 ~ 2.12) | 0.047 |  | 1.62 (1.09 ~ 2.41) | 0.017 |  | 1.65 (1.08 ~ 2.51) | 0.021 |
| P value for trend | 0.046 |  |  | 0.016 |  |  | 0.020 |  |
| Cer (18:1/24:1) (per SD) | 1.31 (1.11 ~ 1.53) | 0.001 |  | 1.39 (1.17 ~ 1.64) | <.001 |  | 1.38 (1.14 ~ 1.66) | <.001 |
| Cer (18:1/24:1) (tertiles) |  |  |  |  |  |  |  |  |
| T1 | 1.00 (Reference) |  |  | 1.00 (Reference) |  |  | 1.00 (Reference) |  |
| T2 | 1.22 (0.84 ~ 1.78) | 0.289 |  | 1.27 (0.86 ~ 1.86) | 0.229 |  | 1.25 (0.84 ~ 1.87) | 0.269 |
| T3 | 1.66 (1.14 ~ 2.41) | 0.008 |  | 1.84 (1.24 ~ 2.72) | 0.002 |  | 1.72 (1.12 ~ 2.66) | 0.014 |
| P value for trend | 0.008 |  |  | 0.002 |  |  | 0.013 |  |

Model1: Crude

Model2: Adjusted for sex, age, BMI, previous PCI, diabetes mellitus, smoking

Model3: Adjusted for sex, age, BMI, previous PCI, diabetes mellitus, smoking, CKD, TG, LDL-C, HDL-C, Ca2+, P, antiplatelet agents use and statins use.

Abbreviations: BMI, body mass index; TG, triglyceride; HDL-C, high density lipoprotein cholesterol; LDL-C, low density lipoprotein cholesterol; CKD, chronic kidney disease; Ca^2+^, serum calcium; P, serum phosphorus; CI, confidence interval; OR, odds ratio; SD, standard deviation.

**Figure S1. Flowchart**

**Figure S2. Spearman correlation heatmap of clinical variables, CAC grade, and plasma ceramide species.**

Color scale indicates the direction and strength of Spearman correlations (ρ, −1 to 1), with warm colors representing positive correlations and cool colors representing negative correlations. Significance codes: ***p* < 0.001; **p* < 0.01; *p* < 0.05. Ceramide concentrations were log₁₀-transformed before analysis.

**Abbreviations:** BMI, body mass index; FBG, fasting blood glucose; TG, triglyceride; TC, total cholesterol; LDL-C, low-density lipoprotein cholesterol; HDL-C, high-density lipoprotein cholesterol; HbA1c, glycated hemoglobin; Ca²⁺, serum calcium; P, serum phosphorus; eGFR, estimated glomerular filtration rate; bSS, baseline SYNTAX score; CAC, coronary artery calcification.;

**Figure S3. Forest plot of subgroup analyses for the association between Cer (18:1/18:0) and coronary artery calcification severity.**

Adjusted odds ratio (OR) and 95% confidence interval (CI) per SD increase in Cer (18:1/18:0) are presented. Covariates adjusted for in the model include sex, age, previous PCI, HTN, DM, smoking, CKD, BMI, TG, LDL-C, HDL-C, Ca^2+^, P, antiplatelet agents use and statins use.

Abbreviations: Cer, ceramide; PCI, percutaneous coronary intervention; HTN, hypertension; DM, diabetes mellitus; CKD, chronic kidney disease.

**Figure S4. Forest plot of subgroup analyses for the association between Cer (18:1/24:1) and coronary artery calcification severity.**

Adjusted odds ratio (OR) and 95% confidence interval (CI) per SD increase in Cer (18:1/24:1) are presented. Covariates adjusted for in the model include sex, age, previous PCI, HTN, DM, smoking, CKD, BMI, TG, LDL-C, HDL-C, Ca^2+^, P, antiplatelet agents use and statins use.

Abbreviations: Cer, ceramide; PCI, percutaneous coronary intervention; HTN, hypertension; DM, diabetes mellitus; CKD, chronic kidney disease.

**Figure S5. Receiver Operating Characteristic Analysis of Plasma Ceramide species for Predicting Moderate/Severe Coronary Artery Calcification**

Abbreviations: AUC, Area Under the Curve.

**Non-ECG-Gated Chest CT Acquisition and AI-Based Coronary Calcium Scoring**

Non–ECG-gated chest CT scans were performed as part of routine clinical care using scanners manufactured by GE Healthcare and Siemens. Patients were scanned in the supine position from the thoracic inlet to the diaphragm. Acquisition parameters were as follows: slice thickness, 1.0 mm; tube voltage, 120 kV; tube current, automatic modulation; pitch, 1.2–1.75; and field of view (FOV), 340–360 mm.

For non–ECG-gated coronary artery calcium scoring (CACS), an artificial intelligence–based chest CT algorithm (uAI Discover® Nongated CACS, United Imaging Intelligence) was applied. The algorithm consisted of three main steps: (1) segmentation of the cardiac region using a first-level 3D convolutional neural network (CNN) to provide the input for subsequent calculations; (2) segmentation of coronary calcified lesions using a second-level 3D CNN, with assignment of each calcified region to the corresponding coronary artery branch (LM, LAD, LCX, RCA); and (3) calculation of the Agatston score, volume score, and mass score for each coronary branch and for the total coronary tree.

The Agatston score was calculated as the product of calcification density and lesion area. Density was scored based on CT attenuation values: 130–199 HU = 1, 200–299 HU = 2, 300–399 HU = 3, and ≥400 HU = 4. This score was then multiplied by the calcified lesion area (mm²). The final total Agatston score was obtained by summing across all CT slices and coronary branches.

**An illustrative case is provided below:**

A 75-year-old female underwent non-contrast cardiac CT (Panels A-C) for quantitative coronary artery calcium (CAC) assessment, revealing extensive calcification with a total Agatston score of 1513 (severe).

**The table summarizes calcium burden across major coronary territories:**

| Coronary Artery | Volume  (mm³) | Equivalent Mass  (mg/CaHA) | Agatston score | Calcification Severity |
| --- | --- | --- | --- | --- |
| LM | 69 | 12 | 73 | Mild |
| LAD | 654 | 147 | 771 | Severe |
| LCX | 503 | 99 | 564 | Severe |
| RCA | 79 | 13 | 105 | Moderate |
| Total | 1305 | 271 | 1513 | Severe |

**NOTE:**

Coronary artery calcium (CAC) scoring classifies calcification severity into four categories based on Agatston score: No calcification (0), Mild calcification (1–100), Moderate calcification (101–400), and Severe calcification (>400). Abbreviations: LM, Left Main Coronary Artery; LAD, Left Anterior Descending Artery; LCX, Left Circumflex Artery; RCA, Right Coronary Artery.
